# Dual PROTACs Versus Dual Inhibitors in Oncology: A Medicinal Chemistry and Linker Design Perspective

**DOI:** 10.3390/ph19081146

**Published:** 2026-07-24

**Authors:** Nicolò Bisi, Abdallah Hamze

**Affiliations:** Department of Chemistry, Université Paris-Saclay, CNRS, BioCIS, Bat. Henri Moissan, 17, Av. des Sciences, 91400 Orsay, France

**Keywords:** dual inhibitors, dual PROTACs, oncology, polypharmacology, targeted protein degradation

## Abstract

**Background/Objectives**: Cancer resistance, pathway redundancy, and compensatory signaling challenge traditional therapies. Dual target strategies address this by engaging two disease-relevant proteins within a single molecule. This review compares classical dual inhibitors with dual proteolysis-targeting chimeras (dual PROTACs) to evaluate the therapeutic advantages of degradation over occupancy. **Methods:** We examine oncology target pairs featuring documented examples of both dual inhibitors and dual PROTACs. The biological rationale for co-targeting is analyzed alongside a comparative assessment of their chemical frameworks, focusing heavily on the synthetic strategies, length, and structure of linkers required for dual PROTAC ternary complex formation. **Results:** While dual inhibitors rely on active-site occupancy, dual PROTACs leverage the ubiquitin–proteasome system for catalytic target elimination. Transitioning from dual inhibition to dual degradation in most cases (>85%) enhances antitumor efficacy, extends duration of action, and overcomes resistance mutations. Optimizing linker design remains the critical factor in balancing the simultaneous degradation kinetics of two distinct proteins. **Conclusions:** Dual PROTACs provide distinct advantages over traditional inhibitors by completely destroying target proteins rather than merely blocking them. This comparison offers a practical entry point and actionable synthetic strategies for medicinal chemists designing multi-target protein degraders.

## 1. Introduction

**Figure 1:**
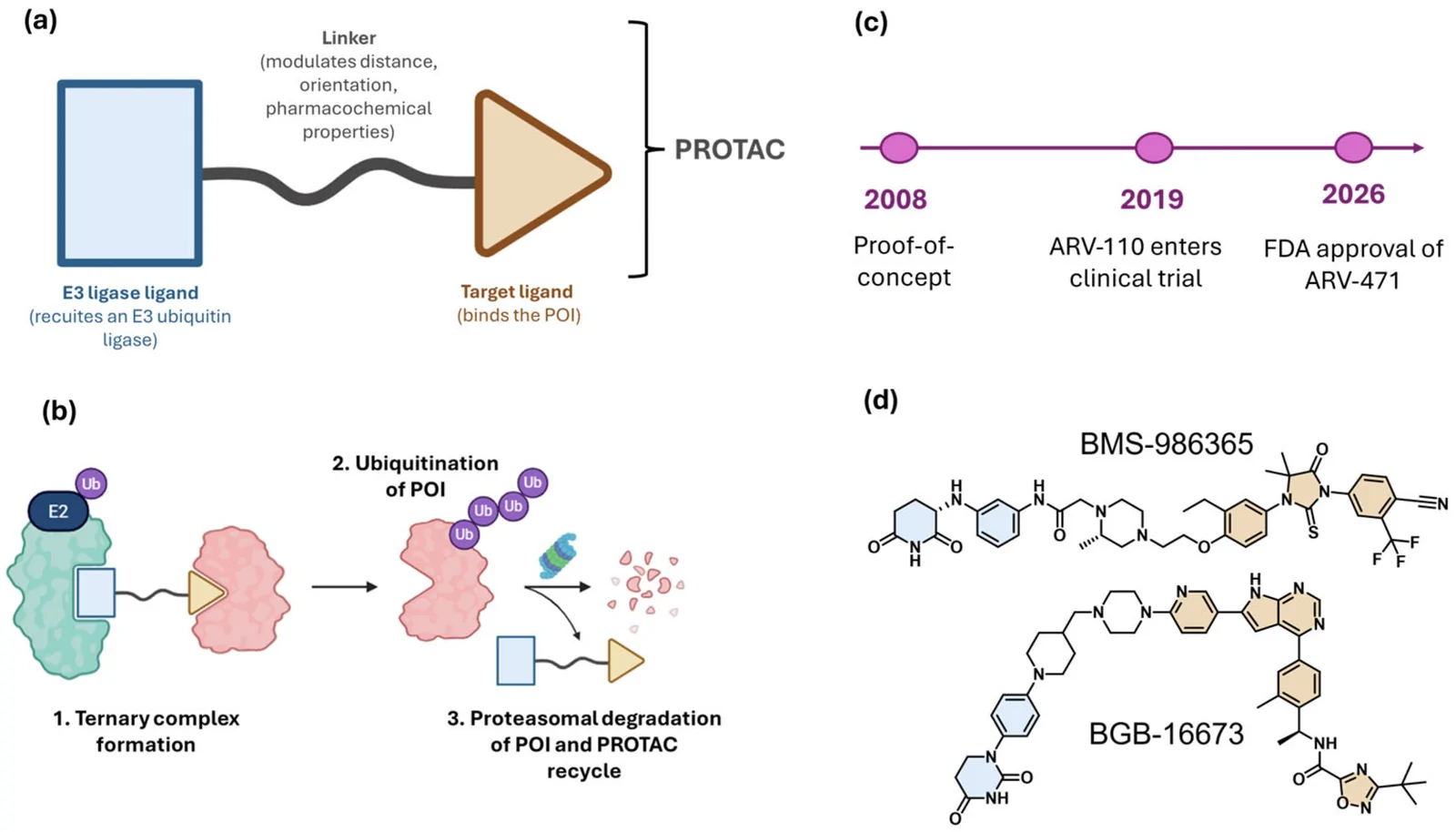
Modular design, catalytic mechanism, and clinical evolution of PROTACs. (**a**,**b**) PROTACs are heterobifunctional degraders that form a ternary complex to induce target protein polyubiquitination and subsequent proteasomal degradation, leaving the intact chimera free to recycle. (**c**) Since the first small-molecule proof-of-concept in 2008, the field has rapidly advanced from initial clinical trials in 2019 to the FDA approval of ARV-471 in 2026 [6,11,12]. (**d**) Chemical structures of advanced Phase III candidates BMS-986365 and BGB-16673, color-coded by target ligand (orange), linker (black), and E3 ligase recruiter (blue).

Proteolysis-targeting chimeras (PROTACs) have emerged as a transformative modality in chemical biology and drug discovery, enabling the selective degradation of disease-relevant proteins rather than their transient inhibition [1]. PROTACs are heterobifunctional molecules composed of three key elements: (i) a ligand that binds the protein of interest (POI), (ii) a ligand that recruits an E3 ubiquitin ligase, and (iii) a linker connecting these two moieties [2]. Upon simultaneous binding to the POI and the E3 ligase, PROTACs induce the formation of a ternary complex that facilitates ubiquitination of the target protein, leading to its subsequent degradation by the ubiquitin–proteasome system (Figure 1) [1,2,3]. This event-driven pharmacology distinguishes PROTACs from conventional occupancy-driven inhibitors and enables sub-stoichiometric activity, catalytic turnover, and the potential to overcome resistance mechanisms [4]. Consequently, PROTAC technology has attracted considerable interest as a new therapeutic strategy across a broad range of diseases, particularly in oncology.

To date, more than 30 PROTACs are under evaluation in clinical trials (mostly Phase I and II), predominantly in oncology indications such as lymphoma and breast cancer [5]. A major milestone for the field was reached with the approval of vepdegestrant (ARV-471; marketed as VEPPANU) by the FDA in 2026 for the treatment of adults with ESR1-mutated, ER-positive/HER2-negative advanced or metastatic breast cancer following endocrine therapy [6]. Two other compounds have now entered Phase III clinical trials: BMS-986365 (also known as CC-94676; an androgen receptor degrader developed by Bristol Myers Squibb, currently in the Phase III rechARge trial for metastatic castration-resistant prostate cancer) [7,8]; and BGB-16673 (catadegbrutinib; a BTK degrader developed by BeOne Medicines, in Phase III trials for relapsed/refractory chronic lymphocytic leukemia and small lymphocytic lymphoma) [9,10].

The vast majority of the developed PROTACs are based on a single-target warhead, meaning that they are designed to induce the degradation of a single protein of interest (POI) [2]. While this approach has proven effective, its therapeutic benefit may be compromised by the emergence of resistance mechanisms, including mutations in the target protein, activation of compensatory signaling pathways, and tumor heterogeneity [5]. Building upon this paradigm, dual PROTACs have recently emerged as an extension of targeted protein degradation strategies [5,13,14,15]. These heterobifunctional systems are designed to simultaneously degrade two distinct protein targets within a single molecular framework, thereby enabling the modulation of multiple disease-relevant pathways. By targeting complementary or synergistic proteins, dual degraders may enhance therapeutic efficacy, reduce the likelihood of resistance, and provide broader biological activity than conventional single-target degraders. However, dual PROTAC development is far from straightforward. Medicinal chemists often face tough selectivity trade-offs, where the molecule might degrade one target perfectly but completely miss the other, or accidentally destroy unintended proteins. There is also very little animal or pharmacokinetic data available yet to prove they actually work in a living body. Ultimately, because these molecules are so large and complex, they struggle with poor absorption and metabolic instability, creating steep roadblocks for clinical translation that scientists must learn to navigate.

Depending on their molecular architecture, dual PROTACs can be classified into three main categories: (i) true dual target degraders, which incorporate two distinct ligands directed against different POIs; (ii) degraders based on promiscuous ligands, capable of engaging and degrading multiple related proteins, such as members of the bromodomain and extra-terminal BET family; and (iii) multivalent or trivalent PROTACs, in which branched architectures enable simultaneous or cooperative interactions with multiple proteins or E3 ligases [5].

The concept of simultaneously engaging two biological targets is not new. Before the development of degraders, scientists focused on the development of dual inhibitors [16]. These are single small molecules capable of modulating two distinct biological targets, a concept long explored in medicinal chemistry to address the complexity of multifactorial diseases [16,17]. These compounds typically rely on either shared pharmacophoric features or hybrid scaffolds combining two inhibitory motifs. One of the earliest clinically successful examples is lapatinib (Tykerb^®^), the first dual inhibitor of epidermal growth factor receptor (EGFR/ErbB1) and human epidermal growth factor receptor (HER2/ErbB2) tyrosine kinases, approved by the FDA in 2007 for the treatment of HER2-positive metastatic breast cancer [18]. Its ability to simultaneously suppress two closely related yet functionally distinct oncogenic drivers within the ErbB family exemplifies the shared pharmacophoric approach to dual inhibition. Here, a single scaffold is accommodated within the highly homologous ATP-binding pockets of both receptors [19]. A second paradigmatic case is sorafenib (Nexavar^®^), originally identified as a Raf kinase inhibitor [20]. It was subsequently found to inhibit multiple additional kinases, including VEGFR-1, -2, and -3, PDGFRβ, FLT-3, c-Kit, and RET receptor tyrosine kinases. Approved by the FDA for advanced renal cell carcinoma, sorafenib was the first targeted therapy approved for this cancer in more than a decade. It subsequently received approval for hepatocellular carcinoma and differentiated thyroid cancer as well [20]. More recently, duvelisib (Copiktra^®^) became the first approved dual inhibitor of PI3K-d and PI3K-g isoforms, both of which are expressed in normal and malignant B-cells [21]. Approved in September 2018 for adult patients with relapsed or refractory CLL/SLL after at least two prior therapies, duvelisib also received accelerated approval for relapsed or refractory follicular lymphoma after at least two prior systemic therapies [22]. Taken together, these examples illustrate that dual inhibitors have been successfully translated into the clinic across a range of cancer types. They demonstrate that simultaneous modulation of two biological targets by a single small molecule is not only achievable but can also constitute a therapeutically meaningful strategy.

**Figure 2:**
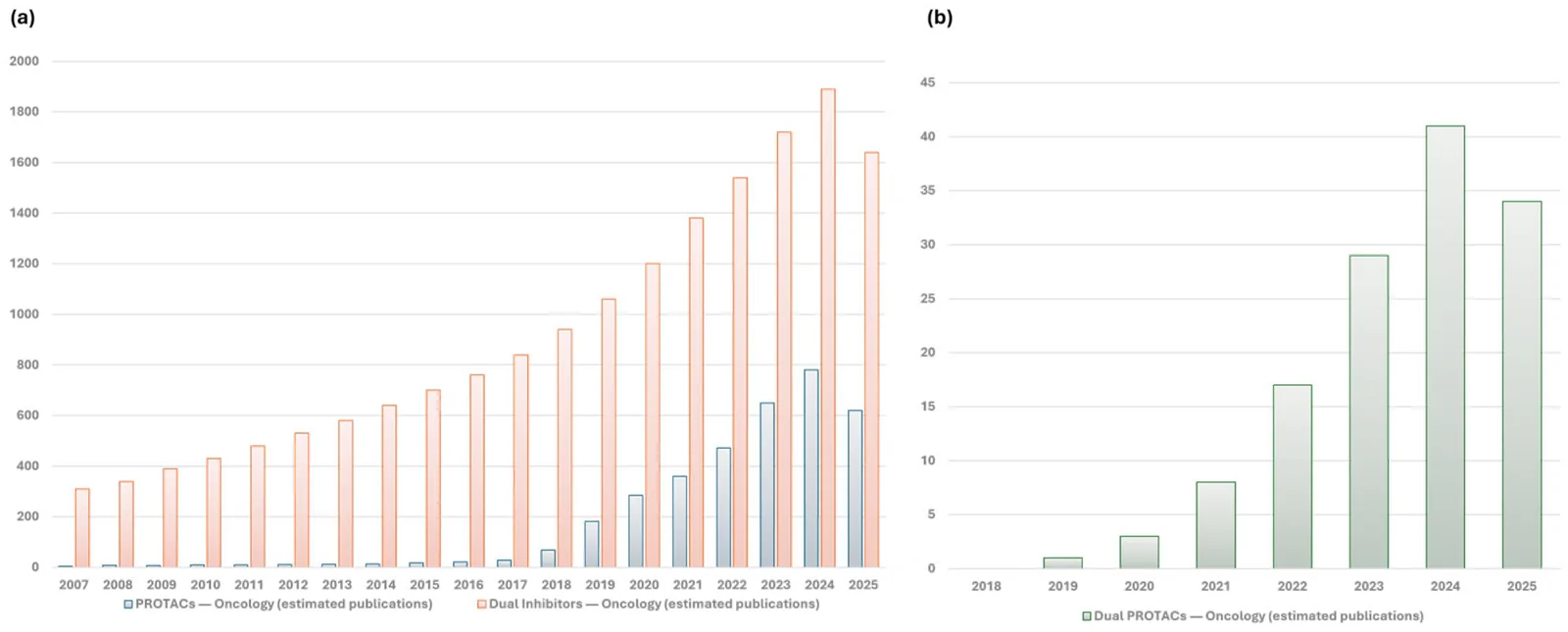
Annual number of oncology-focused publications indexed in PubMed and Scopus (2007–2025). (**a**) Comparative output for the PROTAC field as a whole (blue) and for dual inhibitors (red). (**b**) Subset analysis restricted to dual target PROTACs designed to simultaneously degrade two distinct protein targets (green).

Dual PROTACs and dual inhibitors share the concept of polypharmacology, which recognizes that multifactorial diseases such as cancer may be more effectively addressed by molecules capable of modulating multiple targets [23]. However, these two strategies differ fundamentally in their mechanisms of action, structural complexity, and translational profiles. To appreciate the current state of each field, Figure 2 plots the annual number of oncology-focused publications indexed in PubMed and Scopus between 2007 and 2025.

Panel (a) underlines a contrast in research maturity. Dual inhibitors have maintained a growing publication output throughout this period, while the PROTAC field remained largely confined to academic proof-of-concept studies until 2018. After this year, it underwent exponential growth coinciding with the first clinical translations of targeted protein degraders. Panel (b) narrows the analysis to dual PROTACs specifically. We can see that a meaningful output emerges only from 2021 onward, when Zheng et al. reported the first canonical dual PROTAC degrading EGFR and PARP [24].

Dual inhibitors benefit from well-established optimization frameworks, favorable physicochemical properties, and synthetic scalability that aligns with medicinal chemistry workflows [16]. In contrast, dual PROTACs offer a distinct mode of action based on the simultaneous catalytic degradation of two protein targets. This approach eliminates not only the target enzymatic activity, but also their non-catalytic functions, including scaffolding roles and protein–protein interactions [5]. As a result, targeted protein degradation (TPD) may provide mechanistic advantages that cannot be achieved through occupancy-driven inhibition alone.

**Figure 3:**
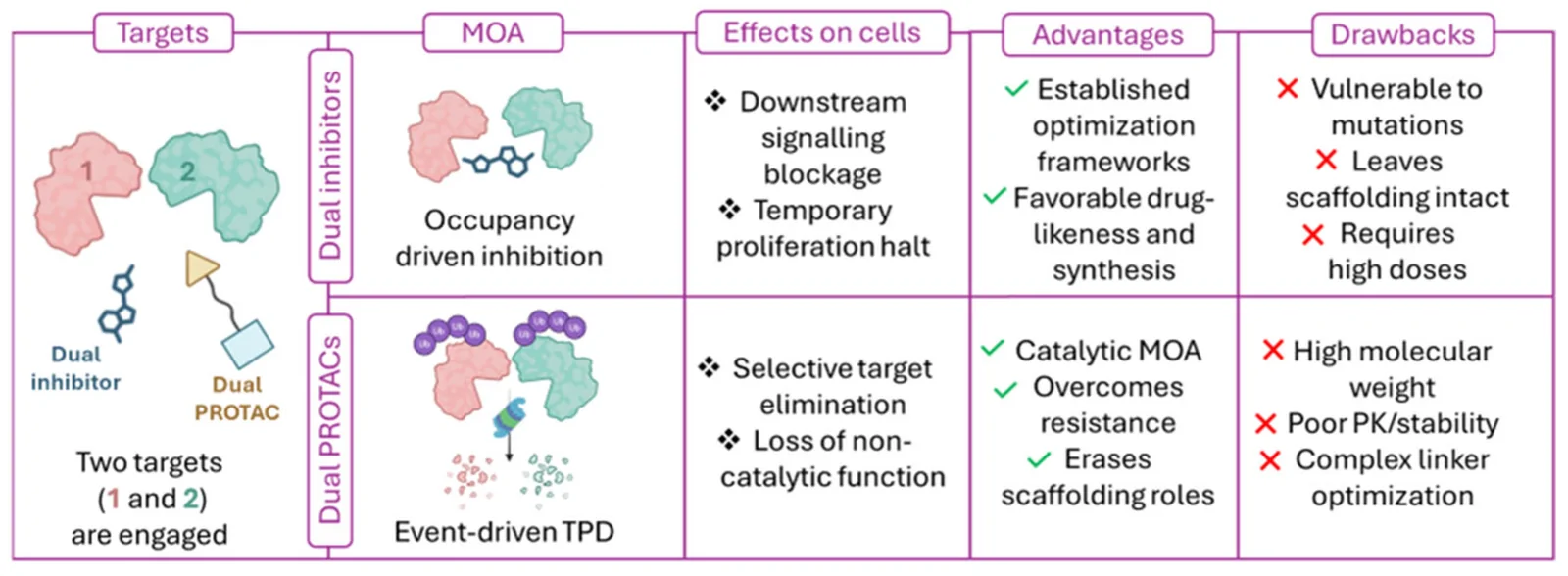
Comparative overview of dual inhibitors and dual PROTACs. Schematic illustration summarizing the structural engagement of dual targets, contrasting mechanisms of action (MOA), cellular effects, and their respective therapeutic advantages and drawbacks. TPD: Targeted Protein Degradation, PK: pharmacokinetics.

These advantages are, however, accompanied by design challenges. Dual PROTACs generally exhibit higher molecular weights, increased structural complexity, and an additional layer of optimization associated with linker design. Linker composition, length, and geometry profoundly influence ternary complex formation, cooperativity, and stability, which ultimately determine degradation efficiency and selectivity [25,26,27,28,29,30]. The main characteristics of dual inhibitors and dual PROTACs, as well as their advantages and drawbacks, are summarized in Figure 3.

The present review provides a comparative analysis of dual PROTACs and dual inhibitors across key development parameters, including drug-likeness, metabolic stability, and pharmacokinetic properties. Its primary objective is to identify the context in which each approach offers the greatest therapeutic potential and to highlight their value as complementary rather than competing strategies. Given that linker architecture represents the principal structural feature distinguishing dual PROTACs from both mono-PROTACs and dual inhibitors, particular attention will be devoted to the linker designs and synthetic strategies reported in the dual PROTAC literature. Through this analysis, we seek to provide medicinal chemists entering the field with practical guidance on linker design, synthesis, and optimization, thereby facilitating the rational development of future dual degraders.

## 2. Results and Discussion

### 2.1. Dual PROTACs vs. Dual Inhibitors

To compare traditional occupancy-driven pharmacology (dual inhibitors) and event-driven targeted protein degradation (TPD, dual PROTACs), we evaluate dual targeting strategies across two distinct categories: homologous protein families and interconnected heterologous networks.

The majority of this discussion focuses on homologous protein families that share conserved structural domains, including cyclin-dependent kinases (CDKs), histone deacetylases (HDACs), BCL-2 family proteins, and CBP/p300 transcriptional coactivators. These target classes represent the most extensively investigated epigenetic and cell-cycle regulatory axes in contemporary oncology [31,32]. Consequently, they provide a robust body of literature that enables a direct comparison between conventional small-molecule inhibition and targeted protein degradation.

To maintain a focused and coherent analysis, specific boundaries have been applied to the selection criteria. While the expanding landscape of TPD includes an array of multi-target systems, an exhaustive cataloging of every emergent heterologous combination lies beyond the scope of this review. Crucially, we explicitly exclude complex, multi-warhead architectures, such as trifunctional “Y-type” PROTACs that tether two separate targeting moieties to a single E3 ligase ligand. This family of compounds are extensively described in other works, notably the review by Liu et al. published in 2024 [5,33,34,35]. Instead, our exploration of heterologous pathways is intentionally restricted to single-warhead bivalent ligands disrupting interconnected networks through a shared binding interface. The structure, the activity, and the targets of the dual PROTACs and dual inhibitors discussed in each part are reported in the corresponding tables.

#### 2.1.1. CDKs

Cyclin-dependent kinases (CDKs) are key regulators of cell-cycle progression and transcription, and their dysregulation is a hallmark of numerous cancers. CDK1, CDK2, CDK4, and CDK6 play essential roles in controlling cell cycle transitions, while CDK7, CDK8, CDK9, CDK12, and CDK13 primarily modulate gene transcription [36]. Overactivation of CDKs usually drives uncontrolled cellular proliferation and tumor growth. Beyond their roles in cell-cycle control, transcription-associated CDKs such as CDK7, CDK9, and CDK12 support oncogenic transcriptional programs and promote cancer cell survival. Consequently, the dependence of many tumors on dysregulated CDK activity has established this protein family as a major therapeutic target, leading to the successful development of selective CDK inhibitors for the treatment of breast cancer and hematological malignancies [37].

Building on this therapeutic rationale, several degraders capable of simultaneously targeting multiple CDK isoforms have been developed through the conjugation of established CDK-binding ligands to cereblon (CRBN)-recruiting moieties. We begin our discussion of dual inhibition and dual degradation strategies with CDK2 and CDK9, two kinases that play complementary roles in cell proliferation and survival. Whereas CDK2 contributes primarily to cell-cycle progression, CDK9 regulates transcriptional elongation through phosphorylation of RNA polymerase II, thereby sustaining the expression of short-lived oncogenic and anti-apoptotic proteins. By sustaining the expression of short-lived oncogenic and anti-apoptotic proteins, including MYC and MCL-1, CDK9 plays a central role in tumor cell survival [38]. Consequently, the simultaneous targeting of CDK2 and CDK9 represents an attractive strategy to disrupt both proliferative and transcriptional survival programs in cancer cells [39].

A few years ago, Zhou and colleagues developed the dual PROTAC F3 by conjugating the CDK inhibitor FN-1501 to the CRBN ligand pomalidomide [40,41]. This PROTAC induced efficient degradation of both CDK2 (DC_50_ = 62 nM) and CDK9 (DC_50_ = 33 nM), and displayed remarkable antiproliferative activity against prostate cancer PC-3 cells (IC_50_ = 120 nM), where it effectively blocked cell-cycle progression at the S and G2/M phases [40]. In parallel, CCT068127 represents a dual CDK2/9 inhibitor derived from optimization of the purine scaffold of seliciclib [42]. The compound inhibits CDK2/cyclin E and CDK9/cyclin T with IC_50_ values of 10 nM and 90 nM, respectively [42]. Importantly, treatment of HT29 colon cancer cells with CCT068127 resulted in decreased tumor-suppressor RB phosphorylation, reduced RNA polymerase II phosphorylation, inhibition of DNA synthesis, and induction of cell-cycle arrest and apoptosis [42]. Its cellular antiproliferative activity is remarkable, with an average GI_50_ of 0.5 μM versus 12 μM across colon cancer and melanoma cell lines. CCT068127 and F3 occupy distinct regions of chemical space, leading to distinct advantages and liabilities in drug development. CCT068127 molecular weight and physicochemical properties remain within Lipinski’s Rule of Five, supporting favorable oral drug-like characteristics. As a PROTAC, F3 places itself in the beyond-Rule-of-Five (bRo5) space [43]. As a result, F3 is expected to display reduced membrane permeability, lower oral bioavailability, and greater metabolic liability relative to CCT068127. From a metabolic perspective, CCT068127 was specifically optimized to overcome the rapid oxidative metabolism associated with its precursor, seliciclib (roscovitine). Structural studies showed a stable binding mode within the CDK2 ATP pocket, including hydrophobic interactions with the gatekeeper residue Phe80, supporting improved metabolic robustness [42]. Conversely, F3 contains several metabolically vulnerable regions, including the methylpiperazine-containing FN-1501 warhead, a flexible linker region, and a pomalidomide-derived CRBN ligand, all of which may contribute to higher intrinsic clearance. Although no direct microsomal stability data are available for F3, its molecular architecture is consistent with the ADME limitations frequently encountered with first-generation PROTACs. From a pharmacological perspective, CCT068127 demonstrated robust cellular efficacy, with approximately 20-fold improved antiproliferative activity over seliciclib and effectively suppressing both RB phosphorylation and RNA polymerase II signaling. F3 instead operates through an event-driven degradation mechanism, whereby transient exposure may be sufficient to induce prolonged CDK2/CDK9 degradation through the ubiquitin-proteasome system. This catalytic mode of action may provide sustained biological effects despite lower systemic exposure. However, no in vivo PK data for F3 has yet been reported, limiting assessment of its translational potential. Finally, CCT068127 benefits from relatively straightforward medicinal chemistry and scalable synthesis based on established trisubstituted purine chemistry. In contrast, F3 requires multistep convergent assembly of the warhead, linker, and E3 ligase ligand, making synthesis and large-scale manufacturing more complex. While CCT068127 presents superior conventional drug-like and pharmacokinetic properties, F3 illustrates the unique mechanistic advantages of targeted protein degradation despite the inherent challenges associated with PROTACs.

TMX-2172 is a potent dual PROTAC derived from the pan-CDK inhibitor dinaciclib, engineered to achieve high degradation selectivity for these two kinases while sparing others like CDK1 and CDK5 [44,45]. In OVCAR8 ovarian cancer cells, TMX-2172 exhibits a significant potency boost, achieving a GI_50_ of 33.1 nM, which outperforms the parent inhibitor’s cellular activity (90 nM average GI_50_) [44,45]. This increased efficacy is driven by its event-driven mechanism, with DC_50_ values reaching 33 nM for CDK2 [45,46]. By utilizing a CRBN-recruiting ligand to physically eliminate the CDK2 “cell cycle engine” and the CDK5 “survival scaffold,” TMX-2172 demonstrates that selective co-degradation can provide a more favorable therapeutic window and deeper cellular response than the broader, more toxic inhibition characteristic of traditional pan-CDK small molecules.

Other groups based PROTACs development on selective CDK inhibitors, as in the case of Jiang and co-workers [47]. The researchers conjugated the dual CDK4/6 inhibitor palbociclib to a CRBN-recruiting ligand to generate BSJ-02-162, which was capable of inducing degradation of both CDK4 and CDK6 [47,48]. BSJ-02-162 displays a DC_50_ of 32 nM and 6.1 nM for CDK4 and CDK6, respectively. This profile reflects the high activity of the parental palbociclib, which showed an IC_50_ of 9 and 15 nM for CDK4/cyclin D1 and CDK6/cyclin D2, respectively. In Mantle Cell Lymphoma (MCL) cell lines, BSJ-02-162 significantly outperforms palbociclib in terms of antiproliferative activity. This superior potency is driven by the “dual threat” mechanism of the PROTAC: it destroys the CDK4/6 proteins and simultaneously degrades the IMiD targets (IKZF1/3), which are vital for MCL survival [49]. In fact, the average GI_50_ for MCL cells (Mino, Granta-519, Geko-1) is 35 nM for BSJ-02-162, showing an 8-fold improvement with respect to palbociclib (280 nM) [47]. Zhao and Burgess developed the dual CDK4/6 PROTACs Pal-pom and Rib-pom using CuAAC “click” chemistry to fuse parent inhibitors (palbociclib and ribociclib, respectively) to a pomalidomide recruiter, significantly enhancing their antiproliferative potency compared to traditional inhibition [48,50]. In MDA-MB-231 breast cancer and A375 melanoma cells, Pal-pom demonstrated superior efficacy with a GC_50_ of 40 nM (a 5-fold improvement over palbociclib), while Rib-pom showed a GC_50_ of 120 nM, reflecting the higher binding affinity of the palbociclib warhead [48,50]. These degraders operate through an event-driven mechanism, achieving DC_50_ values as low as 13 nM for CDK4 and 34 nM for CDK6 for Pal-pom. By physically eliminating the kinases rather than merely occupying their binding sites, these PROTACs induce more robust G1 phase arrest and a deeper suppression of pRb phosphorylation at lower concentrations than their parent compounds, though their high molecular weights (950–1000 Da) present greater challenges for oral bioavailability than the original small-molecule inhibitors.

The research group led by Chinnaiyan and Ding identified compound 7f as a dual CDK12/CDK13 PROTAC built from an analog of the potent parent inhibitor SR-4835 [51,52,53]. 7f exhibits extraordinary degradation efficiency in MDA-MB-231 cells, with DC_50_ values of 2.2 nM (CDK12) and 2.1 nM (CDK13). In the BRCA-deficient TNBC line MFM223, it shows high antiproliferative activity with a GI_50_ of 47 nM, roughly doubling the potency of the parent SR-4835 (100 nM) by inducing a permanent “BRCAness” phenotype through the loss of DNA repair transcripts [51]. Despite its cellular strength, 7f faced significant drug-like hurdles due to poor aqueous solubility. This led to the development of 7b, an optimized derivative with a more balanced lipophilic profile (ClogP = 4.8) [51]. While 7b’s DC_50_ values shifted slightly to 5 nM (CDK12) and 6 nM (CDK13), and its GI_50_ in MFM223 cells moved to 70 nM, it retained nanomolar degradation efficiency while overcoming previous formulation dead-ends. Crucially, 7b demonstrated robust in vivo activity in MDA-MB-231 xenograft models, where doses of 20–30 mg/kg achieved sustained target degradation and significant tumor regression [51]. This transition proves that optimizing the physicochemical properties of a PROTAC is as critical as its catalytic potency for successful translation into systemic therapies.

To finish with, LL-K8-22 is a first-in-class dual degrader of the CDK8–Cyclin C complex, built using the inhibitor BI-1347 as its warhead [54,55]. While BI-1347 is a nanomolar inhibitor of CDK8/19 enzymatic activity, LL-K8-22 achieves more comprehensive cellular control by physically eliminating the kinase and its “undruggable” co-activator, Cyclin C, in MDA-MB-468 TNBC cells. LL-K8-22 exhibits DC_50_ values of 2.5 μM for both targets and provides a 5-fold improvement in antiproliferative activity (GI_50_ of 58 nM) compared to its parent inhibitor. By suppressing oncogenic E2F- and MYC-driven transcriptional programs more persistently, LL-K8-22 illustrates the superior efficacy of an event-driven strategy in eliminating multi-protein complexes driving cancer progression. Interestingly, LL-K8-22 is not a classical PROTAC: in fact, it exploits the hydrophobic tagging (HyT) technology. By mimicking a “misfolded protein” signal, the adamantane tag tricks the endogenous chaperone proteins into recognizing the complex. The chaperones then shuttle the entire CDK8–cyclin C assembly straight to the proteasome for degradation, successfully bypassing the traditional requirement for an E3 ligase [54]. The structure, the activity, and the targets of the dual PROTACs and dual inhibitors discussed in this part are reported in Table 1.

#### 2.1.2. BCL-2 and BCL-xL

As key anti-apoptotic members of the BCL-2 (B-cell lymphoma 2) protein family, the homologous proteins BCL-2 and BCL-xL (B-cell lymphoma-extra-large) are well-established therapeutic targets in oncology [56]. Their aberrant expression drives both tumorigenesis and the development of drug resistance to anticancer therapies, making their simultaneous targeting an attractive therapeutic strategy [57].

The evolution of anti-apoptotic targeting has recently progressed from conventional occupancy-driven inhibition to event-driven targeted protein degradation. The classical dual BCL-2/BCL-xL inhibitor navitoclax (ABT-263, see Table 2 for dosage used in various preclinical models) demonstrated high clinical efficacy by physically blocking the hydrophobic grooves of these anti-apoptotic proteins [58]. This competitive binding displaces pro-apoptotic ‘killer’ proteins (such as BIM), freeing them to permeabilize the mitochondrial membrane and rapidly trigger cancer cell apoptosis [58,59,60].

However, navitoclax clinical translation has been severely hindered by dose-limiting, on-target thrombocytopenia, as platelet survival relies heavily on BCL-xL signaling [61,62]. To decouple anti-tumor activity from this hematological toxicity, Khan and colleagues applied TPD strategies to selectively eliminate BCL-xL [63]. Although the first lead compound, DT2216, was designed as a selective BCL-xL degrader with minimal activity toward BCL-2, subsequent structure-guided optimization of the linker architecture led to the identification of 753b (Table 2), the potent first-in-class dual BCL-2/BCL-xL PROTAC [64,65,66,67]. Compound 753b induced potent degradation of both targets, with DC_50_ values of 6 nM for BCL-xL and 48 nM for BCL-2 [67].

From a drug-likeness and pharmacokinetic perspective, 753b operates well beyond traditional Lipinski’s “Rule of 5” space due to its large, bivalent macromolecular structure (MW: 1641.49). Consequently, whereas navitoclax possesses traditional oral bioavailability, 753b is administered parenterally (intravenously or intraperitoneally) [64,65].

A key advantage of 753b lies in its ability to exploit tissue-specific differences in E3 ligase expression. Human platelets express very low levels of the von Hippel–Lindau (VHL) E3 ligase, limiting the formation of productive ternary complexes and thereby reducing BCL-xL degradation in these cells. As a result, 753b largely preserves platelet viability while maintaining antitumor activity, effectively dissociating BCL-xL targeting from the dose-limiting thrombocytopenia associated with navitoclax [67].

Recent studies show that 753b naturally accumulates in the liver [68]. This liver-specific targeting allows it to act as a highly effective “senolytic”, a drug that selectively destroys aging, damaged cells (senescent cells) that cause tissue degradation [64]. In animal models, 753b successfully cleared these aging cells, slowed the progression of fatty liver disease, and prevented the development of liver cancer (metabolic dysfunction-associated steatohepatitis (MASH)-driven hepatocellular carcinoma) [68]. Importantly, these beneficial therapeutic effects were achieved without causing the severe systemic toxicities seen with earlier small-molecule inhibitors [68].

Building on the structural proof-of-concept established by 753b, subsequent structure-guided optimization of both linker architecture and warhead regions further resulted in the next-generation dual degrader WH244 (Table 2) [66]. This structural fine-tuning enhanced ternary complex cooperativity, driving the DC_50_ values down to 0.6 nM for BCL-xL and 7.4 nM for BCL-2. Consistent with its improved degradation profile, WH244 exhibited superior antiproliferative activity in Jurkat leukemia cells compared with 753b, DT2216, and the parent inhibitor navitoclax, achieving a GI_50_ value of 1.51 nM [66].

Collectively, the progression from navitoclax to DT2216, 753b, and WH244 illustrates how targeted protein degradation can transform a clinically validated yet toxicity-limited pharmacological mechanism into a more selective therapeutic strategy. Although alternative dual targeting approaches have also been explored [69,70], VHL-recruiting dual degraders currently represent one of the most advanced examples of leveraging tissue-specific E3 ligase biology to improve the therapeutic index of BCL-xL-directed therapies.

#### 2.1.3. HDACs

The histone deacetylase (HDAC) family occupies a central hub in cancer pathogenesis. These enzymes regulate chromatin structure and gene expression by removing acetyl groups from histone and non-histone proteins, thereby influencing key cellular processes, including cell-cycle progression, DNA damage repair, apoptosis, and differentiation [71,72]. Consequently, aberrant overexpression or hyperactivation of class I HDAC isoforms (HDAC1, 2, 3, and 8) is frequently associated with tumor initiation, progression, metastasis, and resistance to anticancer therapies, making them attractive targets for therapeutic intervention [71,73,74,75].

The high degree of structural homology among class I HDACs, particularly within their zinc-dependent catalytic domains, has historically favored the development of multitarget inhibitors capable of simultaneously modulating several isoforms [72,76,77,78,79,80]. Indeed, many clinically approved HDAC inhibitors, including vorinostat, panobinostat, and belinostat, display varying degrees of activity across multiple HDAC family members rather than strict isoform selectivity. While such polypharmacology can broaden therapeutic efficacy, it may also contribute to dose-limiting toxicities arising from the inhibition of HDAC isoforms expressed in normal tissues.

More recently, targeted protein degradation has emerged as an alternative strategy to modulate HDAC biology. By eliminating HDAC proteins rather than simply blocking their catalytic activity, HDAC-directed PROTACs have the potential to suppress both enzymatic and non-enzymatic functions, including their participation in transcriptional corepressor complexes. This mechanistic distinction is particularly relevant for class I HDACs, which frequently exert their oncogenic functions as components of large multiprotein assemblies.

This growing trend is illustrated by the VHL-recruiting HDAC1/HDAC3 degrader JPS016 and the HDAC3/HDAC8 degrader YX968 (Table 3) [81,82]. To contextualize the performance of these dual degraders, they should be compared with the pharmacological and pharmacokinetic (PK) profiles of their corresponding parent inhibitors. For instance, the benzamide-based dual HDAC1/HDAC3 inhibitor CI-994 (tacedinaline, Table 3), which served as the warhead for JPS016, exhibits favorable drug-like properties, including an oral bioavailability of approximately 30–40% in preclinical models and a prolonged plasma half-life (approximately 11 h in humans), allowing sustained target occupancy [83,84]. Similarly, traditional hydrazide-class inhibitors, which served as precursors to the dual degrader YX968, possess excellent metabolic stability and low molecular weights (<400 Da), which strictly align with Lipinski’s Rule of 5 and yield excellent cell permeability [82,85].

However, these classical dual inhibitors are intrinsically limited by occupancy-driven mechanics, often demanding high systemic exposure that exacerbates off-target toxicities such as myelosuppression and cardiotoxicity. This is mainly due to a lack of strict isoform selectivity within the highly conserved catalytic pockets of the HDAC family [32,84].

In contrast, dual PROTACs such as JPS016 and YX968 circumvent these pharmacokinetic pitfalls through sub-stoichiometric, event-driven pharmacology, in which sub-stoichiometric target engagement can trigger sustained protein degradation. Although these chimeras inevitably suffer from classic bRo5 liabilities (poor aqueous solubility, high molecular weights, and significantly reduced oral bioavailability), their capacity to form cooperative ternary complexes allows them to achieve isoform selectivity and nanomolar degradation potencies. Specifically, JPS016 effectively drives the simultaneous degradation of HDAC1 (DC_50_ = 550 nM) and HDAC3 (DC_50_ = 530 nM), while YX968 demonstrates exceptional dual degradation of HDAC3 (DC_50_ = 1.7 nM) and HDAC8 (DC_50_ = 6.1 nM) [81,82]. The two PROTACs also showed very good antiproliferative activity against HCT116 (human colon cancer cells) and DLBCL (Diffuse Large B-cell Lymphoma cells) cancer cell lines in the micromolar range (Table 3).

Collectively, these examples illustrate how dual PROTACs can convert clinically relevant but toxicity-limited occupancy-driven inhibitors into degraders with improved isoform selectivity and prolonged pharmacodynamic effects.

#### 2.1.4. CBP and p300

CREB-binding protein (CBP, also known as CREBBP) and its highly conserved paralog p300 (EP300) are twin transcriptional coactivators and lysine acetyltransferases (KATs) that share approximately 61% overall sequence similarity [86]. These multidomain proteins play a pivotal role in cancer pathogenesis by functioning as scaffolding engines that bridge transcription factors with RNA polymerase, while concurrently acetylating histones H3K18 and H3K27 to orchestrate oncogenic enhancer programs like driving MYC expression [86,87]. Dysregulation of CBP/p300 is frequently implicated in acute leukemias and castration-resistant prostate cancer, establishing them as attractive therapeutic targets [86,88].

Traditional small-molecule inhibitors have historically targeted either the bromodomain or the catalytic acetyltransferase domain to suppress specific CBP/p300 functions [89,90,91,92,93]. In contrast, the emergence of heterobifunctional PROTACs provides complete ablation of the entire multi-domain scaffolding framework, eliminating both canonical and noncanonical functions of the proteins [88].

One of the earliest dual CBP/p300 degraders, dCBP-1, was generated by linking the highly selective bromodomain inhibitor GNE-781 to a CRBN-recruiting ligand. (Table 4) [87,94]. GNE-781 displays exceptionally high potency against the CBP and p300 bromodomains (IC_50_ = 0.94 nM and IC_50_ = 2.2 nM, respectively), combined with excellent metabolic stability, low intrinsic clearance (4.4 mL/min in mice), and high oral bioavailability (F = 81% in mice) [94,95]. When converted into dCBP-1 via a flexible linker, the resulting PROTAC preserves sub-nanomolar potency, generating DC_50_ values of 0.8 nM for CBP and 1.9 nM for p300 (Table 4) [87]. However, the dCBP-1 chimera sacrifices these favorable drug-like properties, exhibiting high molecular weight (1028.13 Da) and strict dependency on parenteral or in vitro applications [87]. To overcome these pharmacokinetic boundaries, next-generation chimeras have successfully optimized the bRo5 structural space. JET-209, optimized from the potent catalytic domain inhibitor GNE-207, reaches high degradation potencies, achieving a DC_50_ value of 0.05 nM for CBP and 0.2 nM for p300, alongside a maximum degradation (Dmax) exceeding 95% for both proteins in RS4-11 leukemia lines (Table 4) [96]. Similarly, the highly optimized, orally active PROTAC CBPD-268 achieves an in vitro IC_50_ of 11 nM against CBP and 9.5 nM against p300 [88]. In HiBit assays, CBPD-268 exhibits a DC_50_ of 0.5 nM against CBP and 0.8 nM against p300, which further drops down to a range of 0.01–0.03 nM in androgen receptor-positive (AR+) prostate cancer cells [88]. Crucially, unlike early degraders, CBPD-268 demonstrates outstanding drug-like parameters with an oral bioavailability of 51% in mice, driving complete tumor regression in vivo when orally administered at doses of 0.3–3 mg/kg [88,97]. An alternative strategy has relied on the clinical-stage, orally active inhibitor CCS1477 (inobrodib) as a structural foundation. CCS1477 functions as a potent, selective CBP/p300 bromodomain inhibitor with a K_d_ (binding affinity) of 1.7 nM and over 130-fold selectivity against the off-target BRD4 K_d_ = 222 nM, Table 4) [98]. Structurally building upon CCS1477, Xu et al. developed the CRBN-recruiting dual PROTACs XYD190 and XYD198, which retain high target recognition and completely eliminate the proteins with a DC_50_ around 1.9 nM [99]. While the parent inhibitor CCS1477 exhibits a direct, occupancy-driven profile, the dual PROTACs transform this mechanism into event-driven activity. Consequently, XYD190 and XYD198 exhibit remarkable in vitro antiproliferative activities against the MV4-11 AML cell line (GI_50_ = 1.8 nM and GI_50_ = 0.9 nM, respectively) [99]. This potent in vivo tumor suppression, yielding TGI (tumor-growth inhibition) values of 88% (XYD190) and 93% (XYD198) in MV4-11 xenografts, underscores the profound therapeutic advantage of upgrading standalone domain inhibitors into cooperative, multi-domain degraders [99].

#### 2.1.5. ERα and ARO

The simultaneous targeting of the estrogen receptor alpha (ERα) and the enzyme aromatase (ARO) represents a vital therapeutic approach in hormone receptor-positive breast cancer. This strategy aims to disrupt both the primary oncogenic nuclear transcription factor and the rate-limiting enzyme responsible for estrogen biosynthesis, thereby suppressing estrogen signaling at two complementary levels [100].

Historically, this dual blockade required combination regimens using selective estrogen receptor modulators or downregulators (SERMs or SERDs) alongside traditional aromatase inhibitors (AIs). To combine these distinct mechanisms into a single agent, dual inhibitors resembling norendoxifen were engineered to competitively bind both targets [101]. Norendoxifen, an active metabolite of tamoxifen, and its chemical hybrid derivatives exhibit nanomolar enzymatic aromatase inhibition (IC_50_ = 44 nM), while retaining potent ERα modulation (Table 5) [101]. Owing to their relatively low molecular weight and favorable physicochemical properties, these dual inhibitors remain within conventional rule-of-five chemical space, supporting efficient passive membrane permeability and oral bioavailability. As a result, they represent an attractive example of how classical medicinal chemistry can successfully integrate two complementary endocrine targets within a single small-molecule framework.

However, these occupancy-driven dual inhibitors remain susceptible to clinically relevant resistance mechanisms. In particular, activating ERα mutations such as Y537S and D538G reduce ligand responsiveness and promote constitutive receptor signaling, thereby diminishing the efficacy of endocrine therapies [100].

To overcome these limitations, Zhou and colleagues developed a series of heterobifunctional degraders, evolving from their early ERα-selective PROTAC ZD12 to the dual ERα/ARO degrader compound 18c (Table 5) [102,103]. Compound 18c incorporates a single dual targeting warhead coupled to an E3 ubiquitin ligase ligand via an optimized linker. In doing so, it shifts the pharmacological mechanism from occupancy-driven inhibition to event-driven degradation of both ERα and ARO [103]. Although compound 18c operates in the bRo5 structural space with a high molecular weight (1124.3 Da) that inherently restricts passive cell permeability, its catalytic, event-driven mechanism of action enables remarkable antiproliferative potency against resistant cells [103]. Specifically, compound 18c achieved GI_50_ values of 0.54 µM in wild-type MCF-7, 0.31 µM in endocrine-resistant mutant MCF-7D538G, and 0.075 µM in bypass-resistant MCF-7EGFR lines [103]. Furthermore, the optimized linker structure prevents rapid hepatic metabolism. This ensures high in vivo stability and excellent tumor accumulation, driving significant regression in resistant breast cancer xenografts without the high systemic toxicity of traditional dual inhibitors [103].

### 2.2. Synthesis of Linkers Employed in Dual PROTACs Design

The synthetic preparation of PROTAC linkers dictates both the geometric orientation and the physicochemical properties of the final bifunctional molecule. Depending on the desired degree of conformational flexibility, these spacer units are assembled through a variety of well-established chemical transformations. Here, we will describe the synthesis of the main classes of linkers employed in the dual PROTACs cited in this review.

The synthesis of PROTAC linkers represents a critical aspect of degrader design, as linker architecture directly influences both the geometric arrangement of the target protein and E3 ligase within the ternary complex and the physicochemical properties of the final bifunctional molecule. Parameters such as linker length, rigidity, polarity, and attachment vectors can profoundly affect degradation efficiency, selectivity, cellular permeability, and pharmacokinetic behavior. Consequently, the preparation of linker scaffolds constitutes a key component of modern PROTAC medicinal chemistry. Depending on the desired degree of conformational flexibility and structural complexity, PROTAC linkers can be assembled through a variety of well-established synthetic transformations. In this section, we describe the principal linker classes employed in the dual PROTACs discussed throughout this review, with particular emphasis on their synthetic preparation.

#### 2.2.1. Triazole Linkers

The Copper(I)-catalyzed Azide-Alkyne Cycloaddition (CuAAC) represents a highly efficient, convergent strategy for installing rigid triazole linkers in proteolysis-targeting chimera (PROTAC) synthesis. Owing to its excellent functional-group tolerance, high chemoselectivity, and near-quantitative yields, CuAAC provides a highly convergent strategy for joining complex molecular fragments under mild conditions. Furthermore, the resulting 1,2,3-triazole ring introduces a relatively rigid and metabolically stable structural element that can influence linker conformation and ternary complex formation.

**Scheme 1:**
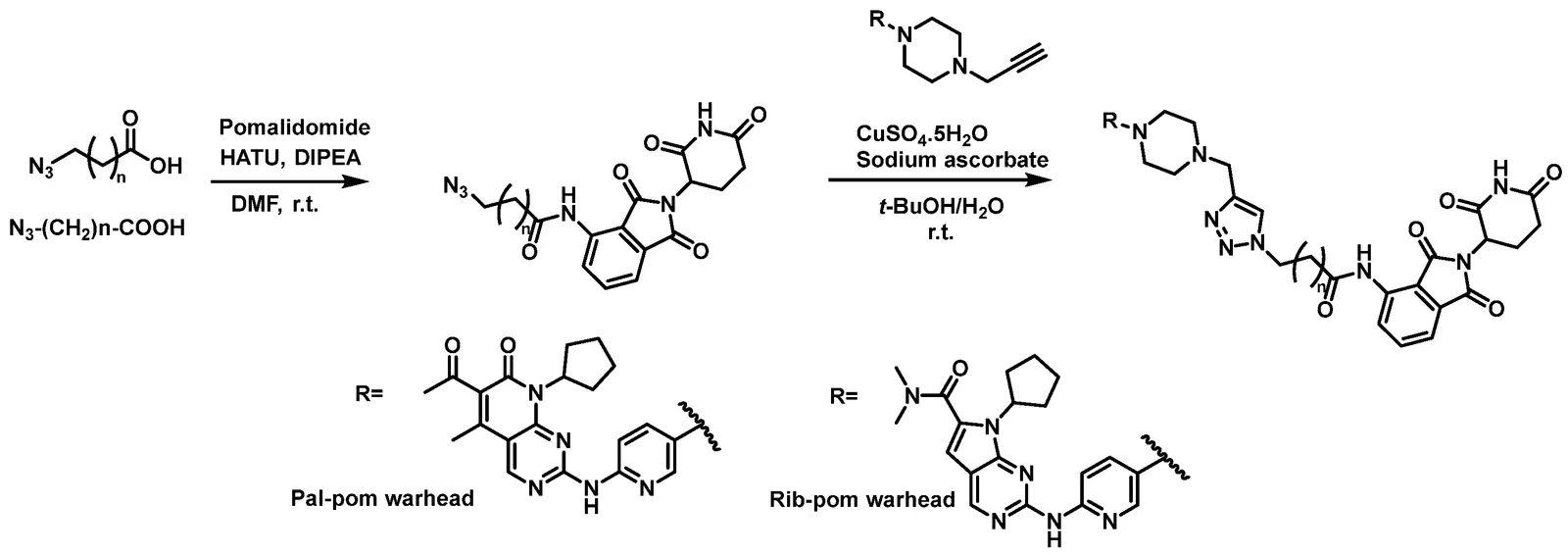
Synthetic strategies to incorporate a triazole linker into a PROTAC structure. As a warhead example, palbociclib and ribociclib structures are reported [48].

This strategy is exemplified by the modular synthesis of the dual CDK4/CDK6 degraders Pal-pom and Rib-pom (Scheme 1), in which azide- and alkyne-functionalized intermediates are coupled in a late-stage CuAAC reaction to generate the final bifunctional degraders.

Structurally, the target protein warhead is functionalized through propargylation of its solvent-exposed piperazine nitrogen using propargyl bromide and K_2_CO_3_ in DMF at room temperature [104]. To prepare the linker-E_3_ ligase recruiter fragment, the 4-amino group of pomalidomide is coupled to commercially available ω-azidoalkanoic acids (N_3_-(CH_2_)_n_-COOH) via HATU-mediated amide bond formation in DMF at room temperature or under mild heating to 60–80 °C. This modular approach readily enables the preparation of linker series differing in length and composition [104,105].

Final convergence is achieved under standard CuAAC conditions employing CuSO_4_·5H_2_O (0.1 eq.) and sodium ascorbate (0.3 eq.) in a 1:1 *t*-BuOH/H_2_O mixture at room temperature or 60 °C [106]. In this particular case, DMSO was added to increase substrate solubility, whereas tris(benzyltriazolylmethyl)amine (TBTA) served as a stabilizing ligand to maintain the catalytically active Cu(I) species and minimize its oxidation [50].

This transformation typically proceeds within 2–12 h, affording the exclusive 1,4-disubstituted 1,2,3-triazole regioisomer in high yields [106]. Beyond its exceptional synthetic efficiency, CuAAC is particularly well suited to parallel and combinatorial synthesis, allowing the rapid optimization of linker length, attachment vectors, and overall degrader architecture [107]. Furthermore, the resulting 1,2,3-triazole ring acts as a metabolically robust amide bioisostere, preserving the spatial arrangement of an amide bond while eliminating its susceptibility to hydrolysis. In addition, triazoles generally exhibit excellent resistance toward both enzymatic degradation and cytochrome P450-mediated oxidation, while their relatively high dipole moment may contribute to improved aqueous solubility compared with purely hydrocarbon linkers [108].

#### 2.2.2. Flexible Alkyl and PEG Linkers

Flexible alkyl and poly(ethylene glycol) (PEG) linkers represent the most widely exploited linker class in PROTAC design. Their conformational flexibility allows efficient sampling of the spatial orientations required for productive ternary complex formation while simultaneously influencing key physicochemical properties such as aqueous solubility, membrane permeability, and pharmacokinetic behavior.

Alkyl linkers are typically introduced as alkylamide spacers extending from solvent-exposed functional groups of the target-binding warhead, most commonly secondary amines or piperazine nitrogen atoms, as illustrated by the dual PROTACs F3, BSJ-02-162, 7f, YX968, and compound 18c. In contrast, PEG linkers consist of one to six ethylene glycol repeat units in most reported PROTACs and are frequently selected to increase linker polarity and aqueous solubility while preserving conformational flexibility [30].

The choice between alkyl and PEG linkers not only influences the physicochemical profile of the degrader but also determines the synthetic strategy used for linker installation, including the order of fragment assembly and the coupling methodology.

**Scheme 2:**
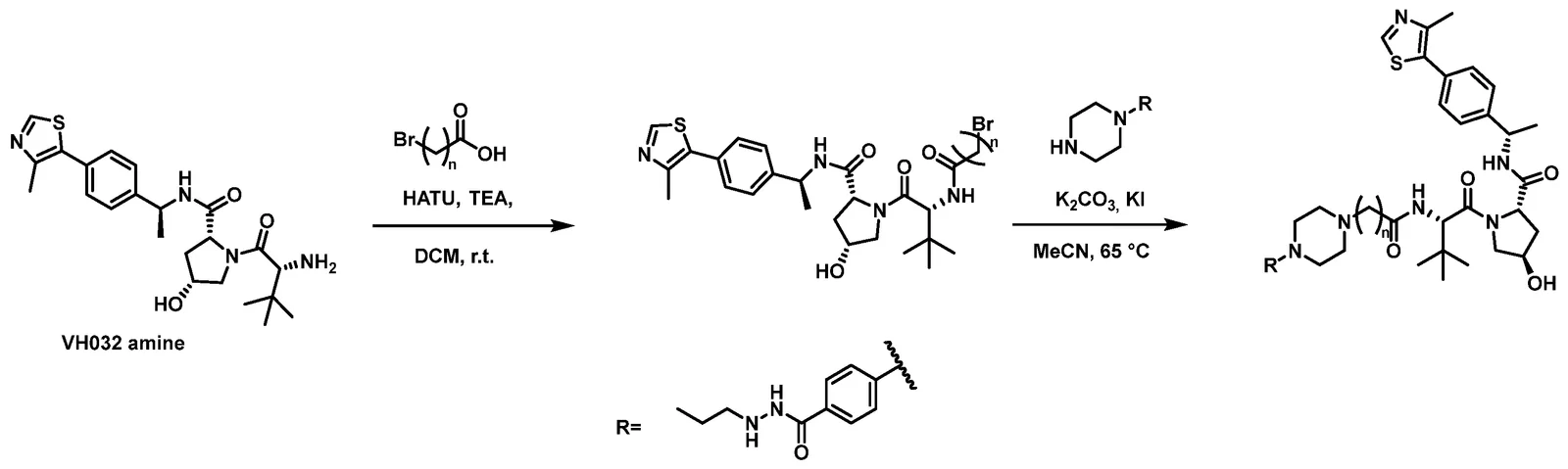
Synthetic strategies to incorporate an alkyl flexible linker into PROTAC structure. For this example, *n* = 7 (PROTAC YX968) [82].

To illustrate the incorporation of a flexible alkyl linker into a dual PROTAC, the synthesis of YX968 provides a representative example (Scheme 2).

In this approach, the commercially available Von Hippel-Lindau (VHL) ligand VH032 amine is employed as the E3 ligase recruiter. Because the hydroxyl group of the hydroxyproline core is sterically hindered, the primary amine is left as the sole accessible, reactive functional group. Linker installation is typically achieved through HATU-mediated amide coupling between VH032 amine and a ω-bromoalkanoic acid (Br-(CH_2_)_n_-COOH) in the presence of triethylamine (TEA) in DCM, usually at room temperature overnight.

The resulting bromide-terminated intermediate subsequently undergoes a nucleophilic substitution (SN_2_) with the nucleophilic functional group of the target-binding warhead, thereby furnishing the final bifunctional degrader. This two-step sequence is operationally simple, highly modular, and readily accommodates systematic variation in alkyl linker length, making it one of the most broadly applicable synthetic strategies for flexible alkyl linkers in PROTAC chemistry [82].

The utility of this linker class was established in the earliest VHL-recruiting PROTACs, where flexible alkyl spacers consistently enabled efficient target degradation. Subsequent structure-guided investigations of VH032 exit vectors further demonstrated that linker attachment geometry strongly influences ternary complex formation and degradation efficiency, establishing flexible alkyl linkers as a benchmark for VHL-based degrader design [109,110].

In kinase optimization campaigns, subtle tuning of the aliphatic span has proven essential for achieving thermodynamic selectivity between homologous targets by stabilizing cooperative ternary complexes [105]. From a medicinal chemistry perspective, pure alkyl chains offer a distinct advantage over equivalent poly(ethylene glycol) (PEG) variants by lowering overall molecular weight (MW), which frequently enhances cell membrane permeability [107]. However, these benefits are often accompanied by reduced aqueous solubility. Furthermore, standard hydrocarbon chains (such as C5 to C6) present a severe in vivo pharmacokinetic liability due to rapid, cytochrome P450-mediated ω- and (ω-1)-oxidation [111]. To mitigate this metabolic vulnerability while preserving lipophilicity, contemporary strategies frequently substitute select methylene units with oxygen atoms (such as replacing -CH_2_-CH_2_-CH_2_- with -CH_2_-O-CH_2_-). This modification interrupts the continuous hydrocarbon chain, decreases susceptibility to oxidative metabolism, and simultaneously improves polarity without introducing the excessive hydrophilicity associated with long PEG sequences. Consequently, short PEG-containing or mixed alkyl/PEG linkers have become among the most widely employed spacer architectures in contemporary PROTAC design [107].

**Scheme 3:**
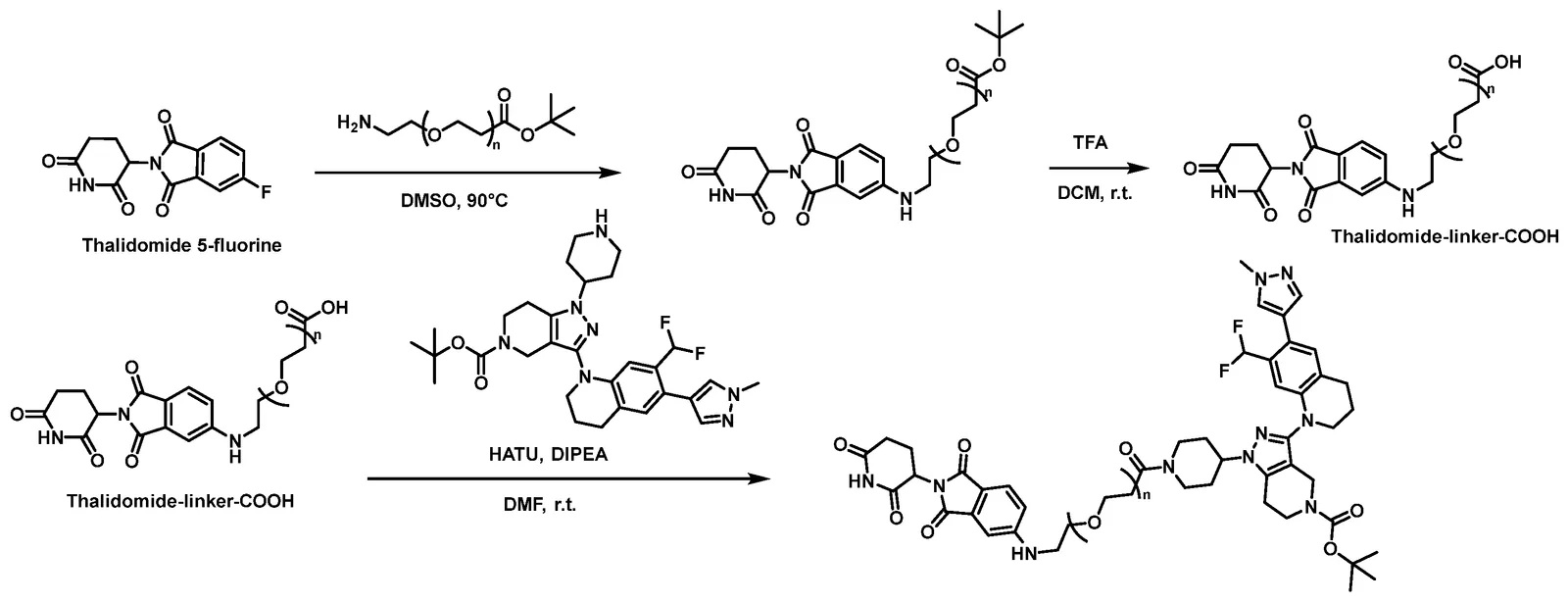
Synthetic strategies to incorporate a PEG flexible linker into PROTAC structure. For this example, *n* = 4 (PROTAC dCBP-1) [87].

As a representative example of PEG linker incorporation into a PROTAC architecture, the synthesis of CBP/p300 degrader dCBP-1 is presented (Scheme 3) [87]. In this strategy, thalidomide 5-fluoride serves as the E3 ligase-recruiting building block and provides a convenient electrophilic handle for the installation of an amine-terminated PEG linker through nucleophilic aromatic substitution (SNAr). Although thalidomide 5-fluoride can be synthesized from 4-fluorophthalic anhydride and 3-aminopiperidine-2,6-dione (glutarimide) [87], it is now commercially available from several suppliers and is therefore commonly employed directly as the starting material. Linker installation is achieved by reacting thalidomide 5-fluoride with the appropriate amino-terminated PEG derivative under heating in a polar aprotic solvent, typically DMSO, which promotes the SNAr reaction (Scheme 3) [87]. Depending on the substrate, the transformation can be performed either under conventional thermal conditions or under microwave irradiation, the latter often reducing reaction times while maintaining comparable or improved isolated yields.

Finally, the tert-butyl ester group is deprotected using trifluoroacetic acid (TFA) in DCM at room temperature to afford the corresponding carboxylic acid. In the final step of this example, the COOH-linker is coupled to the warhead under standard peptide coupling conditions (HATU and DIPEA in DMF) to give the desired PROTAC dCBP-1 [87].

#### 2.2.3. Rigid and Semi-Rigid Linkers

As previously discussed, flexible PEG and alkyl chains are typically deployed to map out the initial spatial landscape required for ternary complex assembly in PROTAC discovery. However, these highly dynamic chains present significant drawbacks. In fact, they require an entropic penalty upon binding, as they must adopt a defined conformation to engage the target protein, and they are also more susceptible to metabolic degradation [107]. To overcome these challenges, rigid and semi-rigid linkers are increasingly employed [26]. A representative example of this design is exemplified by the degrader LL-K8-22, which targets the CDK8–cyclin C complex via a hybrid piperazine–glycine–amide linker assembly [54]. Wang and colleagues anchored a conformationally constrained heterocycle (piperazine) to a semi-flexible spacer (glycine), thereby creating a chimeric motif that balances rigidity and flexibility. This design restricts excessive linker entropy while retaining sufficient adaptability to allow optimal alignment between the target protein and the E3 ligase. As a result, it provides a practical framework for refining early leads into more potent and drug-like degraders.

**Scheme 4:**
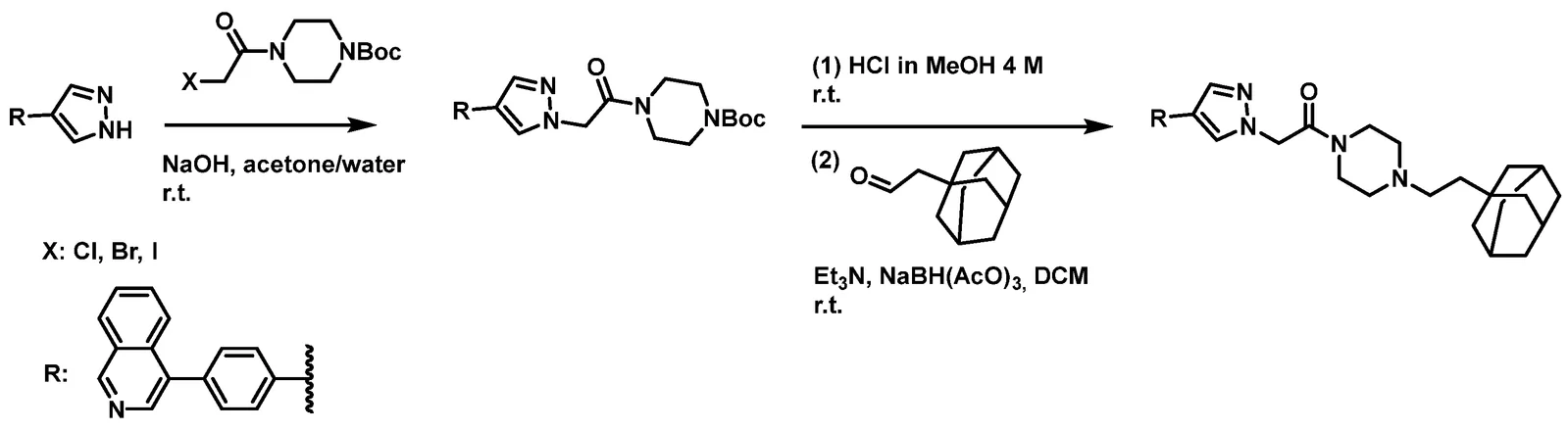
Synthetic strategies to incorporate a piperazine-glycine semi-rigid linker into PROTAC structure. For this example, the PROTAC LL-K8-22 is described [54].

To install the piperazine-glycine linker, the authors adopted a highly efficient, convergent synthetic strategy. Rather than assembling the spacer stepwise, the warhead was directly functionalized at an early stage. The synthesis started with the primary core scaffold derived from the CDK inhibitor BI-1347. The central pyrazole nitrogen underwent a nucleophilic substitution (SN2) reaction with a haloacetamide derivative already bearing a mono-Boc-protected piperazine (Scheme 4). This alkylation step was carried out under mild basic conditions, utilizing NaOH or K_2_CO_3_ in a polar solvent (acetone/water) at room temperature, enabling the incorporation of the piperazine–glycine–amide spacer in a single step.

Following successful installation of the spacer, the intermediate underwent acidic *N*-Boc deprotection. In the reported procedure, HCl in MeOH was employed, but a solution in 1,4-dioxane or TFA in DCM can be used as well. In the final step, the hydrophobic adamantane tag was introduced via a reductive amination sequence. The deprotected piperazine amine intermediate was reacted with an adamantane-alkyl aldehyde derivative in an organic solvent such as DCM. Triethylamine (TEA) was added as a base, and the mixture was stirred briefly to allow in situ formation of the corresponding iminium ion. Subsequently, sodium triacetoxyborohydride NaBH(OAc)_3_ was added as the mild and chemoselective reducing agent, affording the desired PROTAC series.

## 3. Materials and Methods

A systematic literature search was conducted across the PubMed and Scopus databases up to 2026 to identify peer-reviewed articles evaluating dual-targeting small molecules and proteolysis-targeting chimeras (PROTACs) in oncology. Search queries utilized Boolean combinations of terms including (“PROTAC” OR “dual inhibitor”) AND (“cancer” OR “tumor”) to isolate studies reporting definitive chemical structures, synthetic methodologies, and quantitative biological activity metrics (IC_50_, DC_50_). Studies focusing on homologous families (CDKs, HDACs, BCL-2, CBP/p300) and interconnected heterologous networks (ER-α/aromatase) were included, while complex macromolecular layouts like trifunctional “Y-type” architectures were excluded to maintain a strict focus on single-warhead bivalent designs. Data regarding chemical topologies, linker classifications (flexible aliphatic/PEG vs. rigid networks), and synthetic pathways (e.g., CuAAC click chemistry, amide couplings) were extracted and cataloged. All data supporting the findings of this review are thoroughly documented within the manuscript and its figures. No large datasets, animal models, or human studies requiring ethical approval codes were involved in this work. Generative artificial intelligence (GenAI) was utilized during the drafting stage strictly for superficial text editing, formatting, and structural refinement; consequently, no formal GenAI study design disclosures are required under journal guidelines.

## 4. Conclusions and Perspectives

TPD has emerged in recent years as a complementary and transformative paradigm in oncology [34,112,113]. Dual inhibitors and dual PROTACs represent two fundamentally distinct yet complementary strategies for tackling the hallmark challenges of cancer, such as resistance mechanisms, pathway redundancy, and compensatory signaling. Dual inhibitors benefit from well-established optimization frameworks, scalable synthesis, and physicochemical properties consistent with Lipinski’s Rule of Five, facilitating favorable cell permeability and oral bioavailability. However, their reliance on systemic exposure to maintain target occupancy frequently results in off-target toxicity and clinical vulnerability to resistance mutations.

In contrast, dual PROTACs harness the ubiquitin–proteasome system to achieve catalytic, sub-stoichiometric degradation of two distinct targets within a single bifunctional molecule. The recent FDA approval of vepdegestrant in May 2026, alongside multiple candidates advancing into Phase III clinical trials, validates TPD as a validated therapeutic class [6]. By eliminating target proteins rather than merely blocking their active sites, dual PROTACs get rid of both enzymatic and non-canonical scaffolding or protein–protein interaction functions [105]. This distinct mode of action provides a prolonged effect that can outlast systemic exposure and achieves good isoform-selective targeting within highly conserved protein families (such as CDKs, BCL-2, HDACs, and CBP/p300). Also, it can overcome resistance mutations that limit conventional small-molecule inhibitors. Furthermore, as demonstrated by the VHL-recruiting dual BCL-2/BCL-xL degrader WH244, PROTACs can exploit tissue-specific expression of E3 ligases to decouple antitumor efficacy from dose-limiting on-target toxicities, such as thrombocytopenia, commonly associated with dual inhibitors [66]. Despite their strong therapeutic potential, dual PROTACs reside in a region deep within bRo5 chemical space. Their high molecular weights, limited aqueous solubility, reduced passive membrane permeability, and increased metabolic liability present major hurdles for oral drug delivery and pharmacokinetics [107]. Consequently, their successful translation into clinics depends heavily on advanced structural optimization, particularly at the level of linker engineering.

Linker architecture is a critical structural determinant of ternary complex formation, stability, and cooperativity between the E3 ligase and target proteins. Flexible alkyl and PEG-based linkers remain valuable for early-stage screening and spatial mapping. However, they often suffer from high conformational entropy and susceptibility to oxidative metabolism, particularly via cytochrome P450 enzymes [107]. In this context, medicinal chemists are increasingly adopting rigid and semi-rigid linkers, such as triazole-containing motifs or hybrid piperazine–glycine architectures. This strategy aims at reducing conformational flexibility, enhancing metabolic stability, and improving physicochemical properties relevant to membrane permeability [26,29,30,107]. In parallel, emerging degradation strategies that bypass canonical E3 ligase recruitment, such as hydrophobic tagging (HyT), exemplified by the CDK8–cyclin C degrader LL-K8-22, offer additional opportunities to disrupt multi-protein complexes using simpler and lower-molecular-weight design elements [54].

Ultimately, dual inhibitors and dual PROTACs should be viewed not as competing modalities, but as complementary implementations of a broader “proximity pharmacology” paradigm for the modulation of oncogenic targets. Whereas dual inhibitors rely on occupancy-driven suppression of enzymatic function within structurally conserved active sites, dual PROTACs operate through event-driven pharmacology. In this latter scenario, transient productive ternary complex formation is translated into sustained and catalytic target elimination. This fundamental shift from inhibition to induced proximity introduces new design constraints that extend beyond classical structure–activity relationships, placing ternary complex cooperativity, cellular context, and E3 ligase engagement at the center of molecular optimization. Future drug discovery will likely rely on a toolkit of proximity-inducing molecules. By leveraging PROTACs, molecular glues, and emerging lysosomal or hydrophobic-tagging technologies, we can now selectively control protein stability and turnover instead of relying on traditional functional inhibition. In this evolving landscape, dual PROTACs represent a powerful but still emerging class of degraders. Their successful clinical translation will depend on balancing selectivity, cooperativity, and pharmacokinetic properties within these larger, more complex structures.

## Figures and Tables

**Table 1:** Dual inhibitors and dual PROTACs against CDK isoforms.

Strategy	Name	Structure	Target Profile and Potency	Cellular Activity
Bind & Block (Dual Inhibitors)	CCT068127/Seliciclib	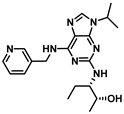	CDK2/cyclin IC_50_ = 10 nM CDK9/cyclinT IC_50_ = 90 nM	Colon cancer and melanoma (avg GI_50_) = 0.5 µM
	Palbociclib	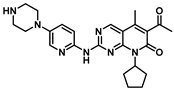	CDK4/cyclin IC_50_ = 9 nM CDK6/cyclin IC_50_ = 15 nM	Mantle cell lymphoma GI_50_ = 280 nM Human melanoma cell (GC_50_) = 200 nM
	SR-4835	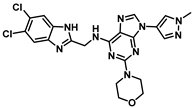	CDK12 IC_50_ = 99 nM CDK13 IC_50_ = 4.9 nM	TNBC—MFM223 GI_50_ = 100 nM
	BI-1347	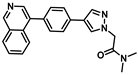	CDK8 IC_50_ = 1.1 nM	MDA-MB-468 TNBC GI_50_ = 290 nM
Bind & Degrade (Dual PROTACs)	F3	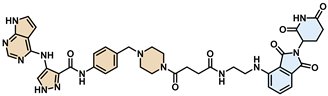	CDK2 DC_50_ = 62 nM CDK9 DC_50_ = 33 nM	PC-3 prostate cancer IC_50_ = 120 nM
	TMX-2172	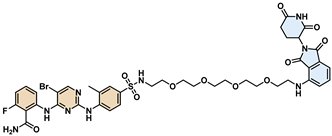	CDK2/cyclinA IC_50_ = 6.5 nM CDK5/p25 IC_50_ = 6.8 nM CDK2 DC_50_ = 33 nM	Ovarian cancer GC_50_ = 33 nM
	BSJ-02-162	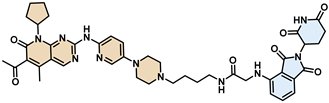	CDK4 DC_50_ = 32 nM CDK6 DC_50_ = 6.1 nM	Mantle cell lymphoma GI_50_ = 35 nM
	Pal-pom	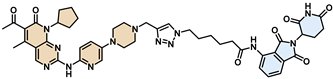	CDK4 DC_50_ = 13 nM CDK6 DC_50_ = 34 nM	Human melanoma cell GC_50_ = 40 nM
	Rib-pom	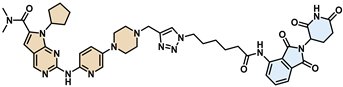	—	Human melanoma cell GC_50_ = 120 nM
	7f	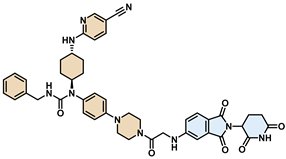	CDK12 DC_50_ = 2.2 nM CDK13 DC_50_ = 2.1 nM	TNBC—MFM223 GI_50_ = 47 nM
	7b	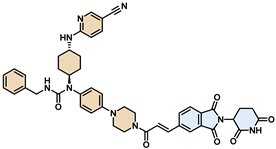	CDK12 DC_50_ = 5 nM CDK13 DC_50_ = 6 nM	TNBC—MFM223 GI_50_ = 70 nM
	LL-K8-22	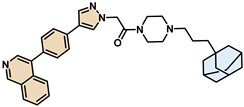	CDK8 DC_50_ = 2.5 µM Cyclin C DC_50_ = 2.5 µM	MDA-MB-468 TNBC GI_50_ = 58 nM

**Table 2:** The structure, IC_50_ and GI_50_ of dual inhibitors and dual PROTACs against BCL-2 and BCL-xL are presented.

Strategy	Name	Structure	Target Profile and Potency	Cellular Activity
Bind & Block (Dual Inhibitors)	Navitoclax (ABT-263)	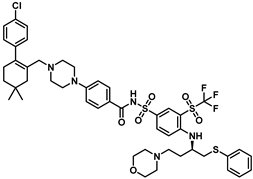	BCL-2 and BCL-xL IC_50_ = 0.5 nM for both proteins	Dosage: 0.001–10.0 µM in SCLC cell lines, 1.0 µM epithelial cancer cell lines, 1–5 µM MDA-MB-231 and MCF-7
Bind & Degrade (Dual PROTACs)	753b	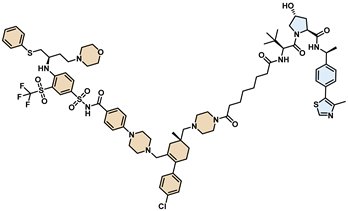	DC_50_ BCL-xL = 6 nM; DC_50_ BCL-2 = 48 nM;	GI_50_ on Kasumi-1 (AML): 59.6 nM, MOLM-1 (AML): 37.0 nM,
	WH244	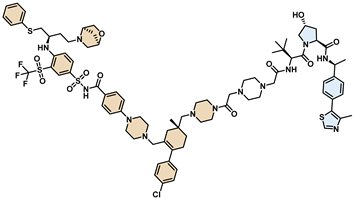	DC_50_ BCL-xL = 0.6 nM; DC_50_ BCL-2 = 7.4 nM;	GI_50_ on MOLM-1/Jurkat: 1.51 nM, H146 (SCLC): 2.10 nM

**Table 3:** Summary of activity values for dual inhibitors and PROTACs active against HDACs.

Strategy	Name	Structure	Target Profile and Potency	Cellular Activity
Bind & Block (Dual Inhibitors)	CI-994 (tacedinaline)	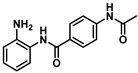	IC_50_ = 0.9 µM (HDAC1), 1.2 µM (HDAC3)	GI_50_ on HCT116 cells: 10 µM
Bind & Degrade (Dual PROTACs)	JPS016	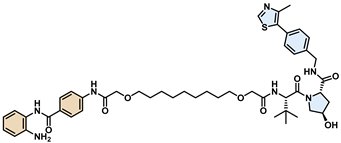	DC_50_ HDAC1 = 550 nM; DC_50_ HDAC3 = 530 nM	GI_50_ on HCT116 cells: 5.3 µM
	YX968	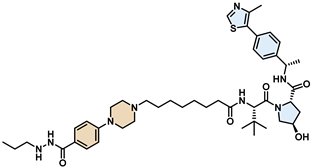	DC_50_ HDAC3 = 1.7 nM; DC_50_ HDAC8 = 6.1 nM	GI_50_ on DLBCL cells: 1.0 µM

**Table 4:** Activity on isolated proteins and on cancer lines relative to dual inhibitors and PROTACs acting on CBP and p300.

Strategy	Compound Name	Structure	Target Profile and Potency	Cellular Activity
Bind & Block (Dual Inhibitors)	GNE-781	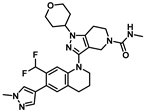	IC_50_ CBP bromodomain = 0.94 nM; IC_50_ p300 bromodomain = 2.2 nM	GI_50_: 31 nM in MOLM-16 cells
	CCS1477 (inobrodib)	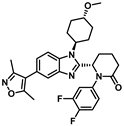	K_d_ CBP/p300 bromodomain = 1.7 nM	GI_50_ = 16 nM—140 nM (hematological and AR+ prostate cancer cell lines)
Bind & Degrade (Dual PROTACs)	dCBP-1	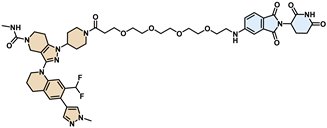	DC_50_ CBP = 0.8 nM; DC_50_ p300 = 1.9 nM	EC_50_ = 13 nM (MOLM-13), EC_50_ = 24 nM (MM.1S myeloma cells)
	JET-209	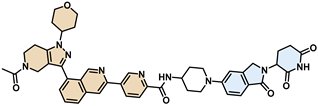	DC_50_ CBP = 0.05 nM; DC_50_ p300 = 0.2 nM	D_max_ > 95% for both targets in RS4-11 leukemia cells
	CBPD-268	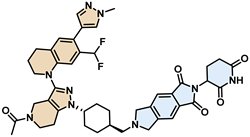	IC_50_ CBP = 11 nM, IC_50_ p300 = 9.5 nM; DC_50_ CBP = 0.5 nM, p300 = 0.8 nM (HiBit); DC_50_ = 0.01–0.03 nM in AR+ prostate cancer cells	GI_50_ = 0.17 nM (MOLM-13); GI_50_ = 2.9 nM (RS4;11)
	XYD190	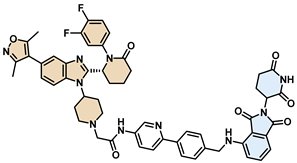	DC_50_ = 1.9 nM (both proteins); GI_50_ (MV4-11 AML) = 1.8 nM;	TGI in MV4-11 xenografts: 88% at 5 mg/kg
	XYD198	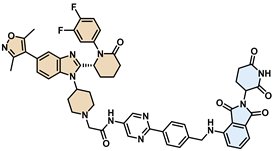	DC_50_ = 0.5 nM (CBP and p300)	GI_50_ (MV4-11 AML) = 0.9 nM; TGI in MV4-11 xenografts: 93% at 5 mg/kg

**Table 5:** List of dual inhibitors and dual PROTACs against Erα and ARO enzymes.

Strategy	Name	Structure	Target Profile and Potency	Cellular Activity
Bind & Block (Dual Inhibitors)	Norendoxifen	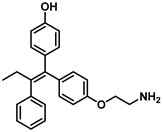	Aromatase IC_50_ = 44 nM	GI_50_ = 5.0 nM (In estrogen-dependent ER+ MCF-7 breast cancer cell models)
Bind & Degrade (Dual PROTACs)	Compound 18c	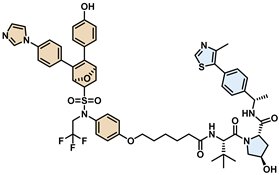	K_i_ ERα = 0.25 μM; IC_50_ Aromatase = 4.6 μM	GI_50_ = 0.54 µM (MCF-7 WT); 0.31 µM (MCF-7 D538G mutant); 0.075 µM (MCF-7 EGFR bypass-resistant)

## Data Availability

No new data were created or analyzed in this study. Data sharing is not applicable to this article.

## Abbreviations

The following abbreviations are used in this manuscript:

ADME	Absorption, Distribution, Metabolism, and Excretion
AI	Aromatase Inhibitor
AML	Acute Myeloid Leukemia
AR/AR+	Androgen Receptor/Androgen Receptor-positive
ARO	Aromatase
ATP	Adenosine Triphosphate
BCL-2	B-cell lymphoma 2
BCL-xL	B-cell lymphoma-extra-large
BET	Bromodomain and extra-terminal
Boc	tert-Butyloxycarbonyl (protecting group)
BRCA	Breast Cancer gene
bRo5	Beyond-Rule-of-Five
BTK	Bruton’s Tyrosine Kinase
CBP/CREBBP	CREB-binding protein
CDK	Cyclin-dependent kinase
CLL	Chronic Lymphocytic Leukemia
CRBN	Cereblon
CuAAC	Copper(I)-catalyzed Azide-Alkyne Cycloaddition
DCM	Dichloromethane
DC50	Half-maximal degrading concentration
DIPEA	N,N-Diisopropylethylamine
DLBCL	Diffuse Large B-cell Lymphoma
Dmax	Maximum degradation
DMF	N,N-Dimethylformamide
DMSO	Dimethyl sulfoxide
DNA	Deoxyribonucleic acid
E2F	E2 Factor (transcription factor family)
EGFR	Epidermal Growth Factor Receptor
EP300/p300	E1A binding protein p300 (transcriptional coactivator)
ER/ERα	Estrogen Receptor/Estrogen Receptor alpha
FDA	Food and Drug Administration
FLT-3	Fms-like tyrosine kinase 3
GC50	Half-maximal growth inhibition concentration
GI50	Half-maximal inhibition of cell growth
HATU	1-[Bis(dimethylamino)methylene]-1H-1,2,3-triazolo[4,5-b]pyridinium 3-oxide hexafluorophosphate
HDAC	Histone deacetylase
HER2	Human Epidermal Growth Factor Receptor 2
HyT	Hydrophobic Tagging
IC50	Half-maximal inhibitory concentration
IMiD	Immunomodulatory imide drug
KAT	Lysine acetyltransferase
MASH	Metabolic dysfunction-associated steatohepatitis
MCL	Mantle Cell Lymphoma
MOA	Mechanism of Action
MW	Molecular Weight
MYC	Myelocytomatosis oncogene
PARP	Poly (ADP-ribose) polymerase
PDGFRβ	Platelet-Derived Growth Factor Receptor beta
PEG	Poly(ethylene glycol)
PI3K-d/PI3K-g	Phosphoinositide 3-kinase delta/gamma isoforms
PK	Pharmacokinetics
POI	Protein of Interest
PROTAC	Proteolysis-targeting chimera
RNA	Ribonucleic acid
SCLC	Small-Cell Lung Cancer
SERD	Selective Estrogen Receptor Downregulator
SERM	Selective Estrogen Receptor Modulator
SLL	Small Lymphocytic Lymphoma
SN1/SN2	Nucleophilic Substitution (unimolecular/bimolecular)
SNAr	Nucleophilic Aromatic Substitution
TBTA	Tris(benzyltriazolylmethyl)amine
TEA	Triethylamine
TFA	Trifluoroacetic acid
TGI	Tumor-growth inhibition
TNBC	Triple-Negative Breast Cancer
TPD	Targeted Protein Degradation
VEGFR	Vascular Endothelial Growth Factor Receptor
VHL	Von Hippel–Lindau
WT	Wild-Type
A549	Human alveolar basal epithelial cell line
COLO205	Human colon cancer cell line
HCC1954	Human breast cancer cell line
HCT116	Human colon cancer cell line
MCF-7	Human breast cancer cell line (Estrogen Receptor positive)
MDA-MB-231/MDA-MB-468	Human triple-negative breast cancer cell lines
MV4-11	Human acute myeloid leukemia cell line
NCI-H1975	Human non-small cell lung cancer cell line
RS4;11	Human acute lymphoblastic leukemia cell line

## References

[1] Li X., Song Y. (2020). Proteolysis-Targeting Chimera (PROTAC) for Targeted Protein Degradation and Cancer Therapy. J. Hematol. Oncol..

[2] Liu Z., Hu M., Yang Y., Du C., Zhou H., Liu C., Chen Y., Fan L., Ma H., Gong Y. (2022). An Overview of PROTACs: A Promising Drug Discovery Paradigm. Mol. Biomed..

[3] Wang C., Zhang Y., Chen W., Wu Y., Xing D. (2024). New-Generation Advanced PROTACs as Potential Therapeutic Agents in Cancer Therapy. Mol. Cancer.

[4] Sun X., Gao H., Yang Y., He M., Wu Y., Song Y., Tong Y., Rao Y. (2019). PROTACs: Great Opportunities for Academia and Industry. Sig. Transduct. Target. Ther..

[5] Liu J., Liu Y., Tang J., Gong Q., Yan G., Fan H., Zhang X., Pu C. (2024). Recent Advances in Dual PROTACs Degrader Strategies for Disease Treatment. Eur. J. Med. Chem..

[6] Tang C., Tian B., Zhang B., Zhang Y., Ke C., Chen M., Wei M., Wang W., Deng X., Zhang Q. (2026). Insights Into Vepdegestrant (ARV-471): The First-in-Class Estrogen Receptor Proteolysis-Targeting Chimera Approaching Food and Drug Administration Approval for Breast Cancer. ChemMedChem.

[7] Nayak S., Norris J.D., Ammirante M., Rychak E., Wardell S.E., Liao D., Toyama B., Kandimalla R., Christoforou A., Tsuji T. (2026). Discovery of BMS-986365, a First-in-Class Dual Androgen Receptor Ligand-Directed Degrader and Antagonist, for the Treatment of Advanced Prostate Cancer. Clin. Cancer Res..

[8] CA071-1000-A Phase 3, Two-Part, Randomized, Open-Label, Adaptive Study Comparing BMS-986365 Versus Investigator’s Choice of Therapy Comprising Either Docetaxel or Second Androgen Receptor Pathway Inhibitor (ARPI), in Participants with Metastatic Castration-Resistant Prostate Cancer (mCRPC)—rechARge—AIOM. http://studiclinici.aiom.it/studi%2dclinici/elenco%2dcompleto%2dstudi%2dclinici/1,510,1.

[9] Wang H., Zhou Q., Li L., Song W., Peng S., Chen X., Xu L., Sumiyoshi T., Jin W., Shen Z. (2024). Bgb-16673, a Selective BTK Degrader, Exhibits Deeper Inhibition of Cancer Cell Signaling Pathways and Better Efficacy in MCL Models. Blood.

[10] BGB-16673-302-A Phase 3, Open-Label, Randomized Study of BGB-16673 Compared to Investigator’s Choice (Idelalisib Plus Rituximab or Bendamustine Plus Rituximab or Venetoclax Plus Rituximab Retreatment) in Patients with Chronic Lymphocytic Leukemia or Small Lymphocytic Lymphoma Previously Exposed to Both BTK and BCL2 Inhibitors. -AIOM. http://studiclinici.aiom.it/studi%2dclinici/elenco%2dcompleto%2dstudi%2dclinici/1,510,1.

[11] Li K., Crews C.M. (2022). PROTACs: Past, Present and Future. Chem. Soc. Rev..

[12] Fan G., Chen S., Zhang Q., Yu N., Shen Z., Liu Z., Guo W., Tang Z., Yang J., Liu M. (2025). Proteolysis-Targeting Chimera (PROTAC): Current Applications and Future Directions. MedComm.

[13] Dutta R., Devarajan A., Talluri A., Das R., Thayumanavan S. (2025). Dual-Action-Only PROTACs. J. Am. Chem. Soc..

[14] Zhang Y., Gu W., Chen W., Zhu J., Fan L., Zhang L., Zhao L., Miao Q. (2025). A Dual-Targeted Molecule for Disease-Activatable Proteolysis Targeting Chimeras and Targeted Radionuclide Therapy of Cancer. J. Am. Chem. Soc..

[15] Chen Y., Xia Z., Suwal U., Rappu P., Heino J., De Wever O., De Geest B.G. (2024). Dual-Ligand PROTACS Mediate Superior Target Protein Degradation in Vitro and Therapeutic Efficacy in Vivo. Chem. Sci..

[16] Pang X., Xu W., Liang J., Liu Y., Li H., Chen L. (2025). Research Progress and Perspectives of Dual-Target Inhibitors. Eur. J. Med. Chem..

[17] Chen J.-F., Guo S.-J., He B., Zheng W., Jiang W.-J., Yuan Z., Xiang Y., Peng C., Xiong W., Shi J.-Y. (2025). Advances of Dual Inhibitors Based on ALK for the Treatment of Cancer. Bioorg. Chem..

[18] Johnston S.R.D., Leary A. (2006). Lapatinib: A Novel EGFR/HER2 Tyrosine Kinase Inhibitor for Cancer. Drugs Today.

[19] Segovia-Mendoza M., González-González M.E., Barrera D., Díaz L., García-Becerra R. (2015). Efficacy and Mechanism of Action of the Tyrosine Kinase Inhibitors Gefitinib, Lapatinib and Neratinib in the Treatment of HER2-Positive Breast Cancer: Preclinical and Clinical Evidence. Am. J. Cancer Res..

[20] Gong L., Giacomini M.M., Giacomini C., Maitland M.L., Altman R.B., Klein T.E. (2017). PharmGKB Summary: Sorafenib Pathways. Pharmacogenet. Genom..

[21] Horwitz S.M., Koch R., Porcu P., Oki Y., Moskowitz A., Perez M., Myskowski P., Officer A., Jaffe J.D., Morrow S.N. (2018). Activity of the PI3K-δ,γ Inhibitor Duvelisib in a Phase 1 Trial and Preclinical Models of T-Cell Lymphoma. Blood.

[22] Rodrigues D.A., Sagrillo F.S., Fraga C.A.M. (2019). Duvelisib: A 2018 Novel FDA-Approved Small Molecule Inhibiting Phosphoinositide 3-Kinases. Pharmaceuticals.

[23] Reddy A.S., Zhang S. (2013). Polypharmacology: Drug Discovery for the Future. Expert Rev. Clin. Pharmacol..

[24] Zheng M., Huo J., Gu X., Wang Y., Wu C., Zhang Q., Wang W., Liu Y., Liu Y., Zhou X. (2021). Rational Design and Synthesis of Novel Dual PROTACs for Simultaneous Degradation of EGFR and PARP. J. Med. Chem..

[25] Gao H., Sun X., Rao Y. (2020). PROTAC Technology: Opportunities and Challenges. ACS Med. Chem. Lett..

[26] Dong Y., Ma T., Xu T., Feng Z., Li Y., Song L., Yao X., Ashby C.R., Hao G.-F. (2024). Characteristic Roadmap of Linker Governs the Rational Design of PROTACs. Acta Pharm. Sin. B.

[27] Li Y., Zhang X., Xie J., Meng D., Liu M., Chang Y., Feng G., Jiang J., Deng P. (2025). Analyzing the Linker Structure of PROTACs throughout the Induction Process: Computational Insights. J. Med. Chem..

[28] Yu S., Hu S., Wang W., Pan C., Zhang H., Zhou C., Liu Y., Li R. (2026). Semi-Rigid Linkers Improve the Pharmacokinetic Properties and Therapeutic Efficacy of BET PROTACs for Cancer Therapy. Eur. J. Med. Chem..

[29] Zografou-Barredo N.A., Hallatt A.J., Goujon-Ricci J., Cano C. (2023). A Beginner’s Guide to Current Synthetic Linker Strategies towards VHL-Recruiting PROTACs. Bioorg. Med. Chem..

[30] Zagidullin A., Milyukov V., Rizvanov A., Bulatov E. (2020). Novel Approaches for the Rational Design of PROTAC Linkers. Explor. Target. Antitumor Ther..

[31] Tomaselli D., Lucidi A., Rotili D., Mai A. (2020). Epigenetic Polypharmacology: A New Frontier for Epi-Drug Discovery. Med. Res. Rev..

[32] Lawless M.W., Norris S., O’Byrne K.J., Gray S.G. (2009). Targeting Histone Deacetylases for the Treatment of Disease. J. Cell Mol. Med..

[33] Rutherford K.A., McManus K.J. (2024). PROTACs: Current and Future Potential as a Precision Medicine Strategy to Combat Cancer. Mol. Cancer Ther..

[34] Zhao L., Zhao J., Zhong K., Tong A., Jia D. (2022). Targeted Protein Degradation: Mechanisms, Strategies and Application. Sig. Transduct. Target. Ther..

[35] Cornu M., Lemaitre T., Kieffer C., Voisin-Chiret A.S. (2025). PROTAC 2.0: Expanding the Frontiers of Targeted Protein Degradation. Drug Discov. Today.

[36] Pellarin I., Dall’Acqua A., Favero A., Segatto I., Rossi V., Crestan N., Karimbayli J., Belletti B., Baldassarre G. (2025). Cyclin-Dependent Protein Kinases and Cell Cycle Regulation in Biology and Disease. Sig. Transduct. Target. Ther..

[37] Ghafouri-Fard S., Khoshbakht T., Hussen B.M., Dong P., Gassler N., Taheri M., Baniahmad A., Dilmaghani N.A. (2022). A Review on the Role of Cyclin Dependent Kinases in Cancers. Cancer Cell Int..

[38] Anshabo A.T., Milne R., Wang S., Albrecht H. (2021). CDK9: A Comprehensive Review of Its Biology, and Its Role as a Potential Target for Anti-Cancer Agents. Front. Oncol..

[39] Bacon C.W., D’Orso I. (2019). CDK9: A Signaling Hub for Transcriptional Control. Transcription.

[40] Zhou F., Chen L., Cao C., Yu J., Luo X., Zhou P., Zhao L., Du W., Cheng J., Xie Y. (2020). Development of Selective Mono or Dual PROTAC Degrader Probe of CDK Isoforms. Eur. J. Med. Chem..

[41] Wang Y., Zhi Y., Jin Q., Lu S., Lin G., Yuan H., Yang T., Wang Z., Yao C., Ling J. (2018). Discovery of 4-((7H-Pyrrolo[2,3-d]Pyrimidin-4-Yl)Amino)-N-(4-((4-Methylpiperazin-1-Yl)Methyl)Phenyl)-1H-Pyrazole-3-Carboxamide (FN-1501), an FLT3- and CDK-Kinase Inhibitor with Potentially High Efficiency against Acute Myelocytic Leukemia. J. Med. Chem..

[42] Whittaker S.R., Barlow C., Martin M.P., Mancusi C., Wagner S., Self A., Barrie E., Te Poele R., Sharp S., Brown N. (2018). Molecular Profiling and Combinatorial Activity of CCT068127: A Potent CDK2 and CDK9 Inhibitor. Mol. Oncol..

[43] Rossi Sebastiano M., Garcia Jimenez D., Vallaro M., Caron G., Ermondi G. (2022). Refinement of Computational Access to Molecular Physicochemical Properties: From Ro5 to bRo5. J. Med. Chem..

[44] Saqub H., Proetsch-Gugerbauer H., Bezrookove V., Nosrati M., Vaquero E.M., de Semir D., Ice R.J., McAllister S., Soroceanu L., Kashani-Sabet M. (2020). Dinaciclib, a Cyclin-Dependent Kinase Inhibitor, Suppresses Cholangiocarcinoma Growth by Targeting CDK2/5/9. Sci. Rep..

[45] Teng M., Jiang J., He Z., Kwiatkowski N.P., Donovan K.A., Mills C.E., Victor C., Hatcher J.M., Fischer E.S., Sorger P.K. (2020). Development of CDK2 and CDK5 Dual Degrader TMX-2172. Angew. Chem. Int. Ed. Engl..

[46] Giarolla J., Holdaway K.A., Nazari M., Aiad L., Sarkar B., Georg G.I. (2025). Targeting Cyclin-Dependent Kinase 2 (CDK2) Interactions with Cyclins and Speedy 1 (Spy1) for Cancer and Male Contraception. Future Med. Chem..

[47] Jiang B., Wang E.S., Donovan K.A., Liang Y., Fischer E.S., Zhang T., Gray N.S. (2019). Development of Dual and Selective Degraders of Cyclin-Dependent Kinases 4 and 6. Angew. Chem. Int. Ed. Engl..

[48] Braal C.L., Jongbloed E.M., Wilting S.M., Mathijssen R.H.J., Koolen S.L.W., Jager A. (2021). Inhibiting CDK4/6 in Breast Cancer with Palbociclib, Ribociclib, and Abemaciclib: Similarities and Differences. Drugs.

[49] Wang L., Yang Z., Li G., Liu Y., Ai C., Rao Y. (2023). Discovery of Small Molecule Degraders for Modulating Cell Cycle. Front. Med..

[50] Zhao B., Burgess K. (2019). PROTACs Suppression of CDK4/6, Crucial Kinases for Cell Cycle Regulation in Cancer. Chem. Commun..

[51] Yang J., Chang Y., Tien J.C.-Y., Wang Z., Zhou Y., Zhang P., Huang W., Vo J., Apel I.J., Wang C. (2022). Discovery of a Highly Potent and Selective Dual PROTAC Degrader of CDK12 and CDK13. J. Med. Chem..

[52] Schmitz M., Kaltheuner I.H., Anand K., Düster R., Moecking J., Monastyrskyi A., Duckett D.R., Roush W.R., Geyer M. (2024). The Reversible Inhibitor SR-4835 Binds Cdk12/Cyclin K in a Noncanonical G-Loop Conformation. J. Biol. Chem..

[53] Houles T., Boucher J., Lavoie G., MacLeod G., Lin S., Angers S., Roux P.P. (2023). The CDK12 Inhibitor SR-4835 Functions as a Molecular Glue That Promotes Cyclin K Degradation in Melanoma. Cell Death Discov..

[54] Wang M., Lin R., Li J., Suo Y., Gao J., Liu L., Zhou L., Ni Y., Yang Z., Zheng J. (2023). Discovery of LL-K8-22: A Selective, Durable, and Small-Molecule Degrader of the CDK8-Cyclin C Complex. J. Med. Chem..

[55] Hofmann M.H., Mani R., Engelhardt H., Impagnatiello M.A., Carotta S., Kerenyi M., Lorenzo-Herrero S., Böttcher J., Scharn D., Arnhof H. (2020). Selective and Potent CDK8/19 Inhibitors Enhance NK Cell Activity and Promote Tumor Surveillance. Mol. Cancer Ther..

[56] Bruncko M., Oost T.K., Belli B.A., Ding H., Joseph M.K., Kunzer A., Martineau D., McClellan W.J., Mitten M., Ng S.C. (2007). Studies Leading to Potent, Dual Inhibitors of Bcl-2 and Bcl-xL. J. Med. Chem..

[57] Newman D.M., Andersen C.L., Cluse L.A., Newbold A., Fraser P., Legg B.W.C., Xu L.K., Mele D.A., Johnstone R.W. (2026). Dual Bcl-2/Bcl-Xl Inhibition via AZD0466 Combines with Immune Checkpoint Blockade to Enhance Anti-Tumour Activity. Cell Death Dis..

[58] Mohamad Anuar N.N., Nor Hisam N.S., Liew S.L., Ugusman A. (2020). Clinical Review: Navitoclax as a Pro-Apoptotic and Anti-Fibrotic Agent. Front. Pharmacol..

[59] Vardon A., Haston S., Hamdi H., Cooksley G., Guiho R., Carvalho D., Carter R., Boult J.K.R., Gharai D., Cloete I. (2025). Navitoclax Acts Synergistically with Irradiation to Induce Apoptosis in Preclinical Models of H3K27M-Altered Diffuse Midline Glioma. Sci. Rep..

[60] Chen E.C., Liu Y., Bell H.L., Galinsky I.A., Luskin M.R., Winer E.S., Stahl M., Vedula R., Volpe V.O., DeAngelo D.J. (2024). Dual Bclxl and BCL2 Inhibition with Navitoclax (NAV), Venetoclax (VEN), and Decitabine (DEC) for Advanced Myeloid Neoplasms (MN): Safety and Biological Activity in a Phase 1 Study. Blood.

[61] Roberts A.W., Seymour J.F., Brown J.R., Wierda W.G., Kipps T.J., Khaw S.L., Carney D.A., He S.Z., Huang D.C.S., Xiong H. (2012). Substantial Susceptibility of Chronic Lymphocytic Leukemia to BCL2 Inhibition: Results of a Phase I Study of Navitoclax in Patients with Relapsed or Refractory Disease. J. Clin. Oncol..

[62] Gandhi L., Camidge D.R., Ribeiro de Oliveira M., Bonomi P., Gandara D., Khaira D., Hann C.L., McKeegan E.M., Litvinovich E., Hemken P.M. (2011). Phase I Study of Navitoclax (ABT-263), a Novel Bcl-2 Family Inhibitor, in Patients with Small-Cell Lung Cancer and Other Solid Tumors. J. Clin. Oncol..

[63] Khan S., Zhang X., Lv D., Zhang Q., He Y., Zhang P., Liu X., Thummuri D., Yuan Y., Wiegand J.S. (2019). A Selective BCL-XL PROTAC Degrader Achieves Safe and Potent Antitumor Activity. Nat. Med..

[64] Jia Y., Han L., Ramage C.L., Wang Z., Weng C.C., Yang L., Colla S., Ma H., Zhang W., Andreeff M. (2023). Co-Targeting BCL-XL and BCL-2 by PROTAC 753B Eliminates Leukemia Cells and Enhances Efficacy of Chemotherapy by Targeting Senescent Cells. Haematologica.

[65] Khan S., Cao L., Wiegand J., Zhang P., Zajac-Kaye M., Kaye F.J., Zheng G., Zhou D. (2024). PROTAC-Mediated Dual Degradation of BCL-xL and BCL-2 Is a Highly Effective Therapeutic Strategy in Small-Cell Lung Cancer. Cells.

[66] Nayak D., Lv D., Yuan Y., Zhang P., Hu W., Nayak A., Ruben E.A., Lv Z., Sung P., Hromas R. (2024). Development and Crystal Structures of a Potent Second-Generation Dual Degrader of BCL-2 and BCL-xL. Nat. Commun..

[67] Lv D., Pal P., Liu X., Jia Y., Thummuri D., Zhang P., Hu W., Pei J., Zhang Q., Zhou S. (2021). Development of a BCL-xL and BCL-2 Dual Degrader with Improved Anti-Leukemic Activity. Nat. Commun..

[68] Yang Y., Jn-Simon N., He Y., Sun C., Zhang P., Hu W., Tian T., Zeng H., Basha S., Huerta A.S. (2025). A BCL-xL/BCL-2 PROTAC Effectively Clears Senescent Cells in the Liver and Reduces MASH-Driven Hepatocellular Carcinoma in Mice. Nat. Aging.

[69] Chang M., Gao F., Chen J., Gnawali G., Wang W. (2022). MDM2-BCL-XL PROTACs Enable Degradation of BCL-XL and Stabilization of P53. Acta Mater. Med..

[70] Wang Z., He N., Guo Z., Niu C., Song T., Guo Y., Cao K., Wang A., Zhu J., Zhang X. (2019). Proteolysis Targeting Chimeras for the Selective Degradation of Mcl-1/Bcl-2 Derived from Nonselective Target Binding Ligands. J. Med. Chem..

[71] Alseksek R.K., Ramadan W.S., Saleh E., El-Awady R. (2022). The Role of HDACs in the Response of Cancer Cells to Cellular Stress and the Potential for Therapeutic Intervention. Int. J. Mol. Sci..

[72] Liu W.-B., Song J., Zhang S.-Y. (2025). A Short Overview of Dual Targeting HDAC Inhibitors. Future Med. Chem..

[73] Aldana-Masangkay G.I., Sakamoto K.M. (2011). The Role of HDAC6 in Cancer. J. BioMed Biotechnol..

[74] Cheshmazar N., Hemmati S., Hamzeh-Mivehroud M., Sokouti B., Zessin M., Schutkowski M., Sippl W., Nozad Charoudeh H., Dastmalchi S. (2022). Development of New Inhibitors of HDAC1–3 Enzymes Aided by In Silico Design Strategies. J. Chem. Inf. Model..

[75] Witt O., Deubzer H.E., Milde T., Oehme I. (2009). HDAC Family: What Are the Cancer Relevant Targets?. Cancer Lett..

[76] Li X., Xu W. (2014). HDAC1/3 Dual Selective Inhibitors—New Therapeutic Agents for the Potential Treatment of Cancer. Drug Discov. Ther..

[77] Daśko M., de Pascual-Teresa B., Ortín I., Ramos A. (2022). HDAC Inhibitors: Innovative Strategies for Their Design and Applications. Molecules.

[78] Cho H., Lee E., Kim J., Shin S., Kim Y.-J., Lee H., Yu J.H., Jeon Y.H., Lee S.W., Lee S.Y. (2024). Discovery of Organosulfur-Based Selective HDAC8 Inhibitors with Anti-Neuroblastoma Activity. Eur. J. Pharm. Sci..

[79] Ji J., Xia M., Xia W., Miao G. (2025). Structural Classification of HDAC Inhibitors and Cancer Treatment Prospects: A Review. Lett. Drug Des. Discov..

[80] Citarella A., Belluti S., Bonanni D., Moi D., Piccinini I., Rinaldi A., Papulino C., Benedetti R., Cuoghi L., Ciolo S.D. (2025). Structure-Based Discovery of Hsp90/HDAC6 Dual Inhibitors Targeting Aggressive Prostate Cancer. J. Med. Chem..

[81] Baker I.M., Smalley J.P., Sabat K.A., Hodgkinson J.T., Cowley S.M. (2023). Comprehensive Transcriptomic Analysis of Novel Class I HDAC Proteolysis Targeting Chimeras (PROTACs). Biochemistry.

[82] Xiao Y., Awasthee N., Liu Y., Meng C., He M.Y., Hale S., Karki R., Lin Z., Mosterio M., Garcia B.A. (2024). Discovery of a Highly Potent and Selective HDAC8 Degrader: Advancing the Functional Understanding and Therapeutic Potential of HDAC8. J. Med. Chem..

[83] Prakash S., Foster B.J., Meyer M., Wozniak A., Heilbrun L.K., Flaherty L., Zalupski M., Radulovic L., Valdivieso M., LoRusso P.M. (2001). Chronic Oral Administration of CI-994: A Phase 1 Study. Investig. New Drugs.

[84] Marsoni S., Damia G., Camboni G. (2008). A Work in Progress: The Clinical Development of Histone Deacetylase Inhibitors. Epigenetics.

[85] Pichlak M., Sobierajski T., Błażewska K.M., Gendaszewska-Darmach E. (2023). Targeting Reversible Post-Translational Modifications with PROTACs: A Focus on Enzymes Modifying Protein Lysine and Arginine Residues. J. Enzym. Inhib. Med. Chem..

[86] Xu L., Xuan H., Shi X. (2025). Dysregulation of the P300/CBP Histone Acetyltransferases in Human Cancer. Epigenomics.

[87] Vannam R., Sayilgan J., Ojeda S., Karakyriakou B., Hu E., Kreuzer J., Morris R., Herrera Lopez X.I., Rai S., Haas W. (2021). Targeted Degradation of the Enhancer Lysine Acetyltransferases CBP and P300. Cell Chem. Biol..

[88] Chen Z., Wang M., Wu D., Bai L., Xu T., Metwally H., Wang Y., McEachern D., Zhao L., Li R. (2024). Discovery of CBPD-268 as an Exceptionally Potent and Orally Efficacious CBP/P300 PROTAC Degrader Capable of Achieving Tumor Regression. J. Med. Chem..

[89] Joy S.T., Henley M.J., De Salle S.N., Beyersdorf M.S., Vock I.W., Huldin A.J.L., Mapp A.K. (2021). A Dual-Site Inhibitor of CBP/P300 KIX Is a Selective and Effective Modulator of Myb. J. Am. Chem. Soc..

[90] Masci D., Puxeddu M., Silvestri R., La Regina G. (2024). Targeting CBP and P300: Emerging Anticancer Agents. Molecules.

[91] Mastracchio A., Lai C., Digiammarino E., Ready D.B., Lasko L.M., Bromberg K.D., McClellan W.J., Montgomery D., Manaves V., Shaw B. (2021). Discovery of a Potent and Selective Covalent P300/CBP Inhibitor. ACS Med. Chem. Lett..

[92] Sasaki M., Kato D., Yoshida H., Shimizu T., Ogiwara H. (2025). Efficacy of CBP/P300 Dual Inhibitors against Derepression of KREMEN2 in cBAF-Deficient Cancers. Cancer Res. Commun..

[93] van Gils N., Martiañez Canales T., Vermue E., Rutten A., Denkers F., van der Deure T., Ossenkoppele G.J., Giles F., Smit L. (2021). The Novel Oral BET-CBP/P300 Dual Inhibitor NEO2734 Is Highly Effective in Eradicating Acute Myeloid Leukemia Blasts and Stem/Progenitor Cells. HemaSphere.

[94] Romero F.A., Murray J., Lai K.W., Tsui V., Albrecht B.K., An L., Beresini M.H., de Leon Boenig G., Bronner S.M., Chan E.W. (2017). GNE-781, A Highly Advanced Potent and Selective Bromodomain Inhibitor of Cyclic Adenosine Monophosphate Response Element Binding Protein, Binding Protein (CBP). J. Med. Chem..

[95] Xiang Q., Wu T., Zhang C., Wang C., Xu H., Hu Q., Hu J., Luo G., Zhuang X., Wu X. (2024). Discovery of a Potent and Selective CBP Bromodomain Inhibitor (Y08262) for Treating Acute Myeloid Leukemia. Bioorg. Chem..

[96] Thomas J.E.I., Wang M., Jiang W., Wang M., Wang L., Wen B., Sun D., Wang S. (2023). Discovery of Exceptionally Potent, Selective, and Efficacious PROTAC Degraders of CBP and P300 Proteins. J. Med. Chem..

[97] Ma M., Li M., Zhang C., Yang Z., Chen X., Lu P., Nie S., Zhang S., Ma S., Qin C. (2024). Discovery of a Highly Potent PROTAC Degrader of P300/CBP Proteins for the Treatment of Enzalutamide-Resistant Prostate Cancer. J. Med. Chem..

[98] Welti J., Sharp A., Brooks N., Yuan W., McNair C., Chand S.N., Pal A., Figueiredo I., Riisnaes R., Gurel B. (2021). Targeting P300/CBP in Lethal Prostate Cancer. Cancer Discov..

[99] Hu J., Xu H., Wu T., Zhang C., Shen H., Dong R., Hu Q., Xiang Q., Chai S., Luo G. (2024). Discovery of Highly Potent and Efficient CBP/P300 Degraders with Strong In Vivo Antitumor Activity. J. Med. Chem..

[100] Chien T.-J. (2021). A Review of the Endocrine Resistance in Hormone-Positive Breast Cancer. Am. J. Cancer Res..

[101] Lv W., Liu J., Skaar T.C., Flockhart D.A., Cushman M. (2015). Design and Synthesis of Norendoxifen Analogues with Dual Aromatase Inhibitory and Estrogen Receptor Modulatory Activities. J. Med. Chem..

[102] Scott J.S., Moss T.A., Balazs A., Barlaam B., Breed J., Carbajo R.J., Chiarparin E., Davey P.R.J., Delpuech O., Fawell S. (2020). Discovery of AZD9833, a Potent and Orally Bioavailable Selective Estrogen Receptor Degrader and Antagonist. J. Med. Chem..

[103] Xin L., Wang C., Cheng Y., Wang H., Guo X., Deng X., Deng X., Xie B., Hu H., Min C. (2024). Discovery of Novel ERα and Aromatase Dual-Targeting PROTAC Degraders to Overcome Endocrine-Resistant Breast Cancer. J. Med. Chem..

[104] Su S., Yang Z., Gao H., Yang H., Zhu S., An Z., Wang J., Li Q., Chandarlapaty S., Deng H. (2019). Potent and Preferential Degradation of CDK6 via Proteolysis Targeting Chimera Degraders. J. Med. Chem..

[105] Yang L., Mao X., Zhang J., Shu J., Huang W., Dong X., Chen Y., Wu M. (2026). Advantages of PROTACs in Achieving Selective Degradation of Homologous Protein Families. Beilstein J. Org. Chem..

[106] Ma N., Wang Y., Zhao B.-X., Ye W.-C., Jiang S. (2015). The Application of Click Chemistry in the Synthesis of Agents with Anticancer Activity. Drug Des. Devel. Ther..

[107] Troup R.I., Fallan C., Baud M.G.J. (2020). Current Strategies for the Design of PROTAC Linkers: A Critical Review. Explor. Target. Anti-Tumor Ther..

[108] Bonandi E., Christodoulou M.S., Fumagalli G., Perdicchia D., Rastelli G., Passarella D. (2017). The 1,2,3-Triazole Ring as a Bioisostere in Medicinal Chemistry. Drug Discov. Today.

[109] Bondeson D.P., Mares A., Smith I.E.D., Ko E., Campos S., Miah A.H., Mulholland K.E., Routly N., Buckley D.L., Gustafson J.L. (2015). Catalytic in Vivo Protein Knockdown by Small-Molecule PROTACs. Nat. Chem. Biol..

[110] Galdeano C., Gadd M.S., Soares P., Scaffidi S., Van Molle I., Birced I., Hewitt S., Dias D.M., Ciulli A. (2014). Structure-Guided Design and Optimization of Small Molecules Targeting the Protein-Protein Interaction between the von Hippel-Lindau (VHL) E3 Ubiquitin Ligase and the Hypoxia Inducible Factor (HIF) Alpha Subunit with in Vitro Nanomolar Affinities. J. Med. Chem..

[111] Johnson A.L., Edson K.Z., Totah R.A., Rettie A.E. (2015). Cytochrome P450 ω-Hydroxylases in Inflammation and Cancer. Adv. Pharmacol..

[112] Hinterndorfer M., Spiteri V.A., Ciulli A., Winter G.E. (2025). Targeted Protein Degradation for Cancer Therapy. Nat. Rev. Cancer.

[113] Sun Y., Li M., Zou Y., Shi B. (2026). Advances in Targeted Protein Degradation for Cancer Immunotherapy. Cell Biomater..

